# Systematics of the ant genus *Proceratium* Roger (Hymenoptera, Formicidae, Proceratiinae) in China – with descriptions of three new species based on micro-CT enhanced next-generation-morphology

**DOI:** 10.3897/zookeys.770.24908

**Published:** 2018-06-04

**Authors:** Michael Staab, Francisco Hita Garcia, Cong Liu, Zheng-Hui Xu, Evan P. Economo

**Affiliations:** 1 University of Freiburg, Faculty of Environment and Natural Resources, Nature Conservation and Landscape Ecology, Tennenbacherstr. 4, 79106 Freiburg, Germany; 2 Freiburg Institute for Advanced Studies (FRIAS), University of Freiburg, Albertstraße 19, 79104 Freiburg, Germany; 3 Biodiversity and Biocomplexity Unit, Okinawa Institute of Science and Technology Graduate University, 1919-1 Tancha, Onna-son, Okinawa, Japan; 4 Key Laboratory of Forest Disaster Warning and Control in Yunnan Province, College of Biodiversity Conservation and Utilization, Southwest Forestry University, Kunming, Yunnan Province 650224, P.R. China

**Keywords:** 3D model, BEF-China, cybertype, Gutianshan National Nature Reserve, subtropical forest, taxonomy, tropical forest, Xishuangbanna

## Abstract

The genus *Proceratium* Roger, 1863 contains cryptic, subterranean ants that are seldom sampled and rare in natural history collections. Furthermore, most *Proceratium* specimens are extremely hairy and, due to their enlarged and curved gaster, often mounted suboptimally. As a consequence, the poorly observable physical characteristics of the material and its scarcity result in a rather challenging alpha taxonomy of this group. In this study, the taxonomy of the Chinese *Proceratium* fauna is reviewed and updated by combining examinations of traditional light microscopy with x-ray microtomography (micro-CT). Based on micro-CT scans of seven out of eight species, virtual 3D surface models were generated that permit in-depth comparative analyses of specimen morphology in order to overcome the difficulties to examine physical material of *Proceratium*. Eight Chinese species are recognized, of which three are newly described: *Proceratium bruelheidei* Staab, Xu & Hita Garcia, **sp. n.** and *P. kepingmai* **sp. n.** belong to the *P. itoi* clade and have been collected in the subtropical forests of southeast China, whereas *P. shohei* **sp. n.** belongs to the *P. stictum* clade and it is only known from a tropical forest of Yunnan Province. *Proceratium nujiangense* Xu, 2006 **syn. n.** is proposed as a junior synonym of *P. zhaoi* Xu, 2000. These taxonomic acts raise the number of known Chinese *Proceratium* species to eight. In order to integrate the new species into the existing taxonomic system and to facilitate identifications, an illustrated key to the worker caste of all Chinese species is provided, supplemented by species accounts with high-resolution montage images and still images of volume renderings of 3D models based on micro-CT. Moreover, cybertype datasets are provided for the new species, as well as digital datasets for the remaining species that include the raw micro-CT scan data, 3D surface models, 3D rotation videos, and all light photography and micro-CT still images. These datasets are available online (Dryad, Staab et al. 2018, http://dx.doi.org/10.5061/dryad.h6j0g4p).

## Introduction

Recent phylogenetic studies have clarified the evolutionary history of ant subfamilies and genera. One higher-level taxon consistently recovered is the subfamily Proceratiinae, which belongs to the poneroid clade (e.g. Brady et al. 2006, Moreau et al. 2006, Blanchard and Moreau 2017, Borowiec et al. 2017). This subfamily currently contains three valid extant genera and one fossil genus with eight fossil and 144 valid extant species, and one fossil genus with four species (Bolton 2018). *Proceratium* Roger, 1863 is the largest genus in the subfamily with 83 extant and six fossil species. However, based on recent molecular phylogenetic results, the monophyly of the genus appears doubtful (Borowiec et al. 2017). While globally distributed, with the majority of species occurring in warm and sufficiently wet climates, the geographic record is very patchy (Baroni Urbani and de Andrade 2003, Hita Garcia et al. 2014, Guénard et al. 2017). Specimens are only rarely collected, usually in leaf litter or soil samples. Colonies typically occur in low densities (but see Masuko 2010) and are small, having usually fewer than 100 workers (but see Onoyama and Yoshimura 2002, Fisher 2005). *Proceratium* have a cryptobiotic lifestyle with hypogeic foraging habits and nesting in leaf litter, rotting wood, top soil, or below stones (Baroni Urbani and de Andrade 2003). As far as it is known, they are specialized predators of the eggs of spiders and other arthropods, which can be stored in large quantities in the nest (e.g. Brown 1957, Brown 1979, Fisher 2005). Notably, some Japanese *Proceratium* species also display larval haemolymph feeding, a behavior otherwise only known from the ‘dracula ant’ subfamily Amblyoponinae (Masuko 1986). However, if this is a typical feature for the whole genus or restricted to a few congeners remains unknown and requires more in-depth natural history data than currently available.

The genus has been comprehensively revised on a global scale by Baroni Urbani and de Andrade (2003). The authors also refined the internal species clades originally erected by Brown (1958) and grouped the genus in eight internal clades that reflect the relationships of a morphology-based phylogeny (Baroni Urbani and de Andrade 2003). Nevertheless, the account on the genus is far from complete, as can be seen in the few single species descriptions (Fisher 2005, Liu et al. 2015a) and regional revisions published since then (Xu 2006, Hita Garcia et al. 2014, 2015). Considering the cryptic lifestyle and extreme rarity in collections, it is very likely that many more species await discovery and formal taxonomic treatment. In China, seven *Proceratium* species from three clades (*P. itoi* clade, *P. silaceum* clade, *P. stictum* clade) have been recorded so far (Xu 2000, Xu 2006, Liu et al. 2015b), albeit the geographic coverage within the country is poor (Guénard and Dunn 2012). The genus is only known from the provinces of Yunnan (six species), Hunan (two species), Zhejiang (one species), and the island of Taiwan (two species). There are no records from the other provinces in south and southeast China where *Proceratium* populations almost inevitably occur.

In the last decade, X-ray microtomography (micro-CT) technology has gained popularity among systematicists and is being increasingly employed in arthropod taxonomy. Micro-CT is a state-of-the-art imaging technology that facilitates the generation of high-resolution, virtual, and interactive three-dimensional (3D) reconstructions of whole specimens or of particular body parts (Hörnschemeyer et al. 2002, Friedrich and Beutel 2008). The virtual nature of such reconstructions enables non-destructive and comprehensive 3D analyses of anatomy and morphology (Faulwetter et al. 2013, Friedrich et al. 2014). Another crucial benefit of micro-CT is its application for virtual dissections and identification of new diagnostic characters (Deans et al. 2012), which has been successfully applied for lepidopterans (Simonsen and Kitching 2014), mayflies (Sartori et al. 2016), and recently ants (Hita Garcia et al. 2017a).

Despite its common usage in invertebrate paleontology, as well as functional and comparative morphology (e.g. Beutel et al. 2008, Berry and Ibbotson 2010, Barden and Grimaldi 2012), until very recently micro-CT was not applied to alpha taxonomy. In the last years, this situation is changing and micro-CT has become a powerful tool to visually enhance and support diagnostic species delimitations, from single species descriptions to revisions. While initially used for polychaetes (Faulwetter et al. 2013), myriapods (Stoev et al. 2013, Akkari et al. 2015), spiders (Michalik and Ramírez 2013), earthworms (Fernández et al. 2014), and flatworms (Carbayo and Lenihan 2016, Carbayo et al. 2016), micro-CT has evolved into a cutting-edge tool increasingly applied for ant taxonomy (Csősz 2012, Fischer et al. 2016, Sarnat et al. 2016, Agavekar et al 2017, Hita Garcia et al. 2017a, 2017b). A detailed and critical assessment of the technology and its applications for ant taxonomy was provided by Hita Garcia et al. (2017b). Another key advantage of applying micro-CT for invertebrate taxonomy is the use of openly available cybertype datasets linked to the original, physical type material (Faulwetter et al. 2013, Akkari et al. 2015, Hita Garcia et al. 2017b).

In this study, we provide a review of the genus *Proceratium* in China, in which we describe three new species: *P. bruelheidei* sp. n. and *P. kepingmai* sp. n. from the *P. itoi* clade from subtropical southeast China and *P. shohei* sp. n. from the *P. stictum* clade from the tropical south of Yunnan Province. The newly available insights from this study suggest that *P. nujiangense* Xu, 2006 is conspecific with *P. zhaoi* Xu, 2000. Thus, we treat *P. nujiangense* syn. n. as a junior synonym of *P. zhaoi*. To distinguish the new species from morphologically similar species, particularly in the *P. itoi* clade, and to ease future identifications, we provide an illustrated key to the Chinese fauna. We also give species accounts for all other valid species and add a locality record for *Proceratium longigaster* Karavaiev, 1935. Like in previous studies (Fischer et al. 2016, Sarnat et al. 2016, Agavekar et al. 2017, Hita Garcia et al. 2017a, 2017b), we continue using and exploring microtomography for ant taxonomy. In order to visually enhance the taxonomic descriptions, we provide still images and 3D videos based on surface volume renderings of micro-CT scans from all Chinese species (except for *P. longmenense* Xu, 2006). Since the treated species are rather hairy, often dirty, and too scarce for any physical specimen manipulations, we also use the 3D reconstructions for virtual in-depth examinations of surface morphology. Furthermore, the complete micro-CT datasets containing the scan raw data, 3D rotation videos, still images of 3D models, and 3D surfaces supplemented by color montage photos are made freely available online (Staab et al. 2018, http://dx.doi.org/10.5061/dryad.h6j0g4p) as cybertypes.

## Materials and methods

### Abbreviations of depositories

The collection abbreviations follow Evenhuis (2018). The material upon which this study is based is located or will be deposited at the following institutions:

**BMNH** The Natural History Museum, London, UK

**CASC** California Academy of Sciences, San Francisco, California, USA

**MHNG** Muséum d’Histoire Naturelle de la Ville de Genève, Geneva, Switzerland

**NIAES** National Institute for Agro-Environmental Sciences, Japan

**NHMB** Naturhistorisches Museum Basel, Switzerland

**OIST** Okinawa Institute of Science and Technology, Onna-son, Japan

**SIZK** Schmalhausen Institute of Zoology, Kiev, Ukraine

**SWFU** Southwest Forestry University, Kunming, Yunnan, PR China

**TARI** Taiwan Agricultural Research Institute, Taichung, Taiwan

**ZMBH** Museum für Naturkunde der Humboldt-Universität, Berlin, Germany

### Specimens and imaging

The material of the new species was collected during recent ecological field work activities of the first author (see e.g. Staab et al. 2014, Staab et al. 2017) and others (see Liu et al. 2016). All available worker specimens were mounted and measured with a Leica M125 stereo microscope under magnification of 80–100×. To compose montage images, we took raw image stacks with a Leica M205C microscope equipped with a Leica DFC450 camera and then assembled montage images with Helicon Focus (version 6) software. Additional material of previously described *Proceratium* species known to occur in China and of Asian species from the three *Proceratium* species clades containing Chinese species (*P. itoi* clade, *P. silaceum* clade, *P. stictum* clade) was also examined (see species and specimen data in Suppl. material 1: Table S1 for non-Chinese species). The other distributional data used for map generation was extracted from Antmaps.org (Janicki et al. 2016, Guénard et al. 2017).

### Measurements and indices

The following measurements (all expressed in mm) and indices are based on Hita Garcia et al. (2014, 2015):

**EL** Eye length: maximum length of eye measured in oblique lateral view.

**HL** Head length: maximum measurable distance from the mid-point of the anterior clypeal margin to the mid-point of the posterior margin of head, measured in full-face view. Impressions on anterior clypeal margin and posterior head margin reduce head length.

**HLM** Head length with mandibles: maximum head length in full-face view including closed mandibles.

**HW** Head width: maximum head width directly above the eyes, measured in full-face view.

**MFeL** Metafemur length: maximum length of metafemur measured along its external face.

**MTiL** Metatibia length: maximum length of metatibia measured along its external face.

**MBaL** Metabasitarsus length: maximum length of metabasitarsus measured along its external face.

**LT3** Abdominal tergum III length: maximum length of abdominal tergum III (=length of abdominal segment III) in lateral view.

**LS4** Abdominal sternum IV length: maximum length of abdominal sternum IV in lateral view.

**LT4** Abdominal tergum IV length: maximum length of abdominal tergum IV in lateral view.

**PeL** Petiolar length: maximum length of the petiolar node in dorsal view including its anterior prolongation.

**PeW** Petiolar width: maximum width of petiole measured in dorsal view.

**SL** Scape length: maximum length of scape shaft excluding basal condyle.

**TL** Total body length: combined length of HLM + WL + PeL + LT3 + LT4.

**WL** Weber’s length: diagonal length of mesosoma in lateral view from the anterior-most point of pronotal slope (excluding neck) to posteroventral margin of propodeal lamella or lobe.

**CI** Cephalic index: HW / HL * 100

**OI** Ocular index: EL / HW * 100

**SI** Scape index: SL / HL * 100

**MFeI** Metafemur length index: MFeL / HW * 100

**MTiI** Metatibia length index: MTiL / HW * 100

**MBaI** Metabasitarsus length index: MBaL / HW * 100

**DPeI** Dorsal petiole index: PeW / PeL * 100

**ASI** Abdominal segment index: LT4 /LT3 * 100

**IGR** Gastral reflexion index: LS4 / LT4

### X-ray micro computed tomography and 3D images

We scanned all Chinese *Proceratium* species, except for *P. longmenense* from which no material was available for micro-CT analysis. For each of the new species, we scanned the holotype worker specimen, whereas for the remainder of the species we either scanned a paratype or non-type specimen, if no type material was available. An overview of scanning parameters and specimens used is provided in Table 1. All micro-CT scans were performed using a Zeiss Xradia 510 Versa 3D X-ray microscope operated with the Zeiss Scout-and-Scan Control System software (version 11.1.6411.17883). 3D reconstructions of the resulting scan raw data were done with the Zeiss Scout-and-Scan Control System Reconstructor (version 11.1.6411.17883) and saved in DICOM file format. Volume renderings, surface mesh generations, virtual examinations and dissections were performed with Amira software (version 6.3.0). Post-processing of mesh data in order to generate clean surfaces was done with Meshlab (version 1.3.3). The methodology for the virtual examinations of 3D surface models, generation of 3D rotation videos, and virtual dissections follow Hita Garcia et al. (2017a). For more details on micro-CT scanning and post-processing workflow pipeline, we refer to the exhaustive descriptions in Hita Garcia et al. (2017a, 2017b).

**Table 1.** Overview of micro-CT scanning data presenting specimen data, scan settings, and voxel sizes for the resulting scans (all specimens are workers and all files are in DICOM format).

Species	Identifier	Type status	Magnification (x)	Exposure (s)	Voxel size (µm)	Source distance (mm)	Detector distance (mm)	Voltage (kV)	Power (W)	Amperage (µA)
*deelemani*	CASENT0790842	non-type	4	1.5	4.826	24.99	10	45.24	3.54	78.14
*kepingmai*	CASENT0790031	holotype	4	1.5	4.359	19.99	11	45.23	3.54	78.3
*bruelheidei*	CASENT0790023	holotype	4	1.5	4.359	19.99	11	45.24	3.55	78.35
*itoi*	OKENT0016142	non-type	4	0.8	3.660	13	11	45.24	3.54	78.29
*japonicum*	CASENT0790834	non-type	4	0.8	3.897	14.99	11	45.24	3.54	78.17
*longigaster*	CASENT0790673	non-type	4	0.8	3.097	11	13	45.24	3.54	78.19
*nujiangense*	CASENT0790672	paratype	4	1.5	2.534	12	19.99	45.24	3.54	78.19
*shohei*	CASENT0717686	holotype	4	1.5	4.193	17.99	11	45.24	3.53	78.05
*zhaoi*	CASENT0790671	paratype	4	1.5	2.252	11	22	45.24	3.54	78.33

### Data availability

All specimens used in this study have been databased and the data are freely accessible on AntWeb (http://www.antweb.org). Each specimen can be traced by a unique specimen identifier attached to its pin. The Cybertype datasets provided in this study consist of the full micro-CT original volumetric datasets (in DICOM format), 3D surface models (in STL and PLY formats), 3D rotation video files (in .mp4 format, see Suppl. material), all light photography montage images, and all image plates including all important images of 3D models for each species. All data have been archived and are freely available from the Dryad Digital Repository (Staab et al. 2018, http://dx.doi.org/10.5061/dryad.h6j0g4p). In addition to the cybertype data at Dryad, we also provide freely accessible 3D surface models of all treated species on Sketchfab (https://sketchfab.com/arilab/collections/proceratium).

## Results

### Synopsis of Chinese *Proceratium* species

#### *Proceratium itoi* clade

*Proceratium bruelheidei* Staab, Xu & Hita Garcia sp. n. [China: Jiangxi, Zhejiang]

*Proceratium itoi* (Forel, 1918) [China: Hunan, Zhejiang; Taiwan; Japan; South Korea; Vietnam]

*Proceratium kepingmai* Staab, Xu & Hita Garcia sp. n. [China: Jiangxi, Zhejiang]

*Proceratium longmenense* Xu, 2006 [China: Yunnan]

*Proceratium zhaoi* Xu, 2000 [China: Yunnan]

= *Proceratium nujiangense* Xu, 2006 [China: Yunnan] syn. n.

#### *Proceratium silaceum* clade

*Proceratium japonicum* Santschi, 1937 [China: Yunnan; Taiwan; Japan]

= *Proceratium formosicola* Terayama, 1985 [Taiwan]

*Proceratium longigaster* Karavaiev, 1935 [China: Hunan, Yunnan, Zhejiang; Vietnam]

#### *Proceratium stictum* clade

*Proceratium shohei* Staab, Xu & Hita Garcia sp. n. [China: Yunnan]

### Identification key to Chinese *Proceratium* species (workers)

This key is partly derived from Baroni Urbani and de Andrade (2003) and Xu (2006).

**Table:** 

1	In profile, petiolar node squamiform and rectangular, high and erect (Fig. 1A)	**2**
–	In profile, petiolar node never squamiform, either low, elongate, and barrel-shaped, or rounded-triangular (Fig. 1B)	**3**
2	In profile, petiolar node clearly narrowing dorsally, broader on the base than on the apex (Fig. 2A); in dorsal view, petiole at least 1.5× wider than long (DPeI ≥155); abundant long appressed shaggy hairs from LT3 project distinctly over the constriction between LT3 and LT4 (Fig. 2A) [China: Hunan, Yunnan, Zhejiang; Vietnam]	***P. longigaster***
–	In profile, petiolar node not or only weakly narrowing dorsally, the base as or almost as broad as the apex (Fig. 2B); even if narrowing, petiole never 1.5× times wider than long in dorsal view (DPeI <150); no shaggy hairs protruding from LT3 over the constriction to LT4, if single longer hairs present, then not shaggy (Fig. 2B) [China: Yunnan; Taiwan; Japan]	***P. japonicum***
3	Anterior clypeal margin with a distinct and broad notch (Fig. 3A); posterodorsal corners of propodeum armed with conspicuous thick and acute teeth (Fig. 3C); head, mesosoma, petiole, and abdominal segment III strongly foveolate (Fig. 22) [China: Yunnan]	***P. shohei***
–	Anterior clypeal margin without a distinct and broad notch (Fig. 3B); posterodorsal corner of propodeum bluntly rounded or angular (Fig. 3D), never with conspicuous teeth as above; head, dorsal mesosoma, petiole, and abdominal segment III usually granulate or punctate (Figs 8, 10, 12, 14, 15)	**4**
4	Frontal carinae weakly developed, short, little diverging above antennal insertions, and with narrow lateral lamellae; dorsal surface of body without erect hairs protruding from dense pubescence (Fig. 4A); smaller species (WL ≤0.80) with shorter legs (MFeI <80, MTiI <65, MBaI <40) [China: Yunnan]	***P. zhaoi***
–	Frontal carinae better developed, long, diverging above antennal insertions, and usually with broad lateral lamellae; dorsal surface of body with erect hairs protruding from dense pubescence (Fig. 4B); larger species (WL >0.95) with longer legs (MFeI >80, MTiI >65, MBaI >50)	**5**
5	In profile, posterodorsal corners of propodeum rounded (Fig. 5A) [China: Hunan, Zhejiang; Taiwan; Japan; South Korea; Vietnam]	***P. itoi***
–	In profile, posterodorsal corners of propodeum angular (Fig. 5B)	**6**
6	Scapes without erect hairs protruding from the dense pubescence; frontal carinae touching each other at their anteriormost level (Fig. 6A), their lateral lamellae relatively narrow, not conspicuously broader above antennal insertions; head (CI 85) and scapes (SI 68) relatively long [China: Yunnan]	***P. longmenense***
–	Scapes with many erect hairs protruding from the dense pubescence; frontal carinae clearly separated at their anteriormost level, not touching each other, their lamellae broad, conspicuously extending laterally above antennal insertions (Fig. 6B); head relatively broad (CI ≥89) and scapes (SI ≤63) relatively short	**7**
7	Propodeal declivity punctured, mostly opaque; frontal furrow conspicuous and darker than anterior cephalic dorsum (Fig. 7A); posterior face of petiolar node in profile steeper than anterior face and about half as long as anterior face (Fig. 7C) [China: Jiangxi, Zhejiang]	***P. kepingmai***
–	Propodeal declivity very shiny, at most superficially punctured; frontal furrow inconspicuous and of same color than anterior cephalic dorsum (Fig. 7B); posterior face of petiolar node in profile as steep as anterior face and less than half as long as anterior face (Fig. 7D) [China: Jiangxi, Zhejiang]	***P. bruelheidei***


**Figure 1.**
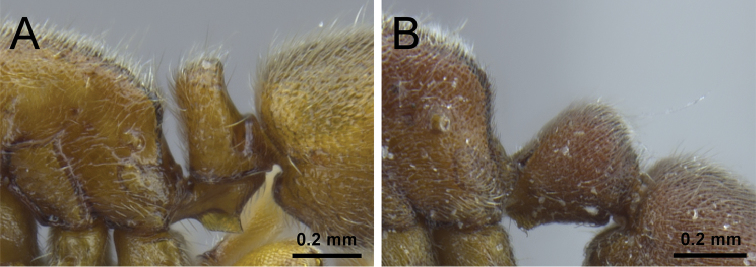
Petiole in profile view. **A** *P. japonicum* (CASENT0790834) **B** *P. itoi* (OKENT0016142).

**Figure 2.**
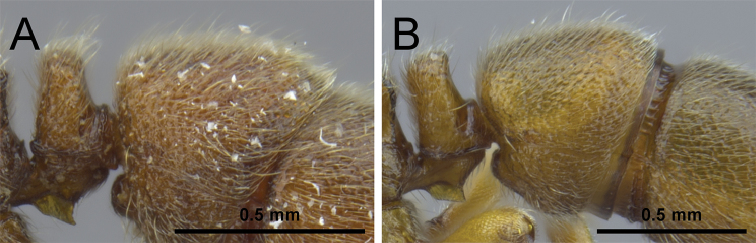
Petiole, abdominal segment III and anterior portion of abdominal segment IV in profile view. **A** *P. longigaster* (CASENT0790673) **B** *P. japonicum* (CASENT0790834).

**Figure 3.**
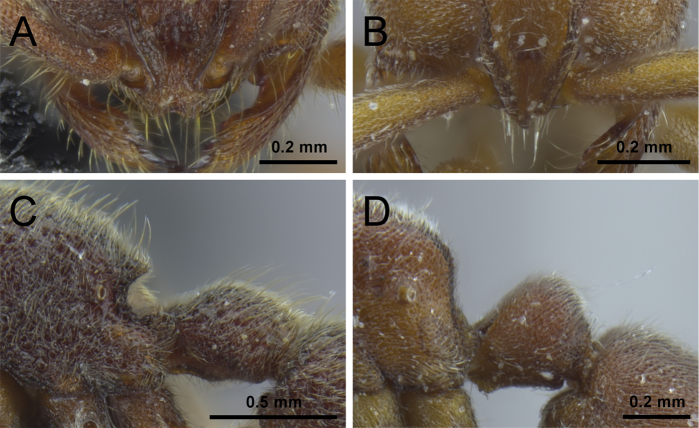
Anterior portion of cephalic dorsum, in full-face view (**A, B**), and propodeum and petiole, in profile view (**C, D**). **A, C** *P. shohei* (CASENT0717686) **B, D** *P. itoi* (OKENT0016142).

**Figure 4.**
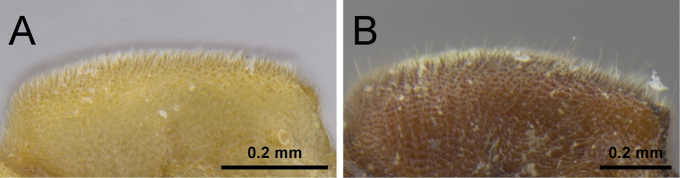
Mesosoma dorsum in profile view. **A** *P. zhaoi* (CASENT0790671) **B** *P. bruelheidei* (CASENT0790023).

**Figure 5.**
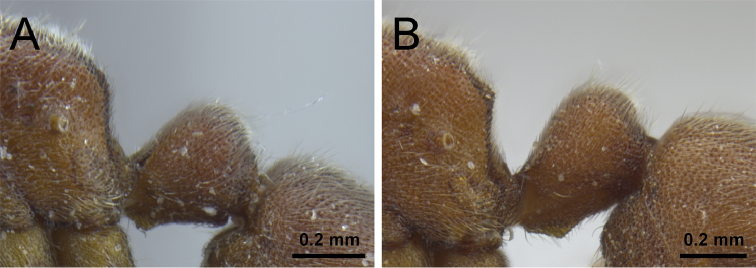
Propodeum and petiole in profile. **A** *P. itoi* (OKENT0016142) **B** *P. kepingmai* (CASENT0790031).

**Figure 6.**
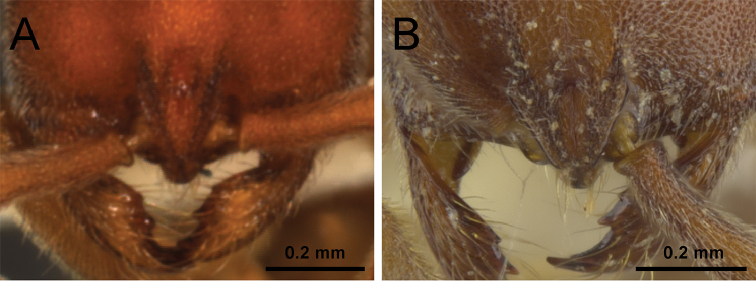
Anterior portion of cephalic dorsum in full-face view. **A** *P. longmenense* **B** *P. bruelheidei* (CASENT0790023).

**Figure 7.**
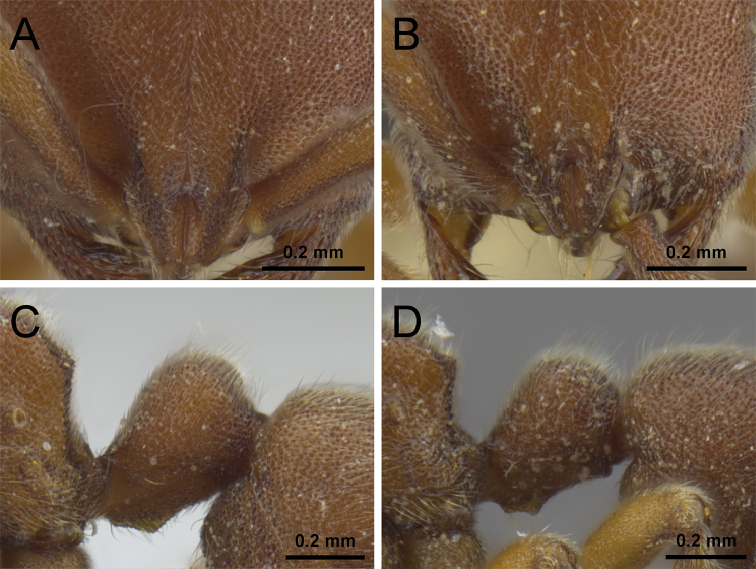
Anterior portion of cephalic dorsum in full-face view (**A, B**) and petiole in profile (**C, D**). **A, C** *P. kepingmai* (CASENT0790031) **B, D** *P. bruelheidei* (CASENT0790023).

### Taxonomic species accounts of Chinese *Proceratium*

#### Proceratium itoi

Taxon classificationAnimaliaORDOFAMILIA

clade

##### Definition.

Workers of this clade can be separated from all other *Proceratium* species by the combination of abdominal sternum IV protruding over the posterior end of abdominal sternum III, petiolar node nodiform, and body sculpture densely granulate to punctate (definition follows Baroni Urbani and de Andrade 2003).

##### Comments.

This clade includes seven species and is restricted to east and southeast Asia. All species except *P. malesianum* de Andrade, 2003 and *P. williamsi* Mathew & Tiwari, 2000 occur in China. The species are morphologically similar, particularly in relative body proportions and indices, but can be safely separated with the identification key presented above. More detailed accounts on species delimitation and biology are reported at the species accounts below.

#### Proceratium bruelheidei

Taxon classificationAnimaliaORDOFAMILIA

Staab, Xu & Hita Garcia sp. n.

http://zoobank.org/D5261D06-D21C-4149-B716-2347DC616BD5

**Figure 8.**
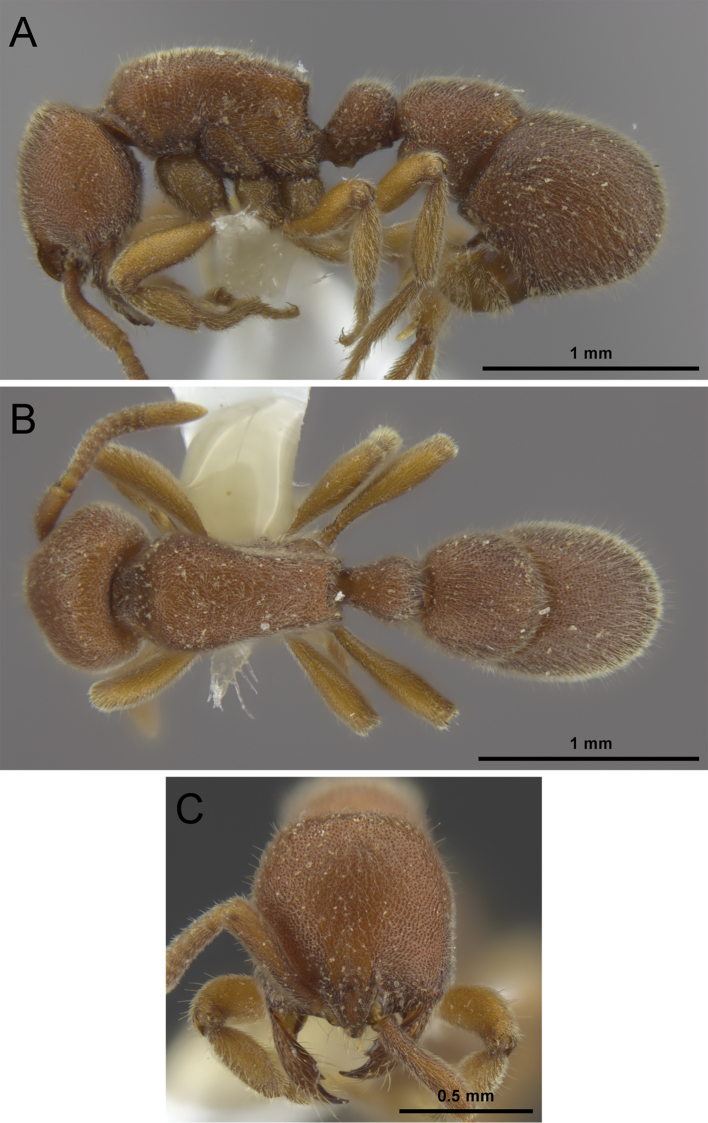
*Proceratium bruelheidei* sp. n. holotype worker (CASENT0790023). **A** Body in profile **B** Body in dorsal view **C** Head in full-face view.

**Figure 9.**
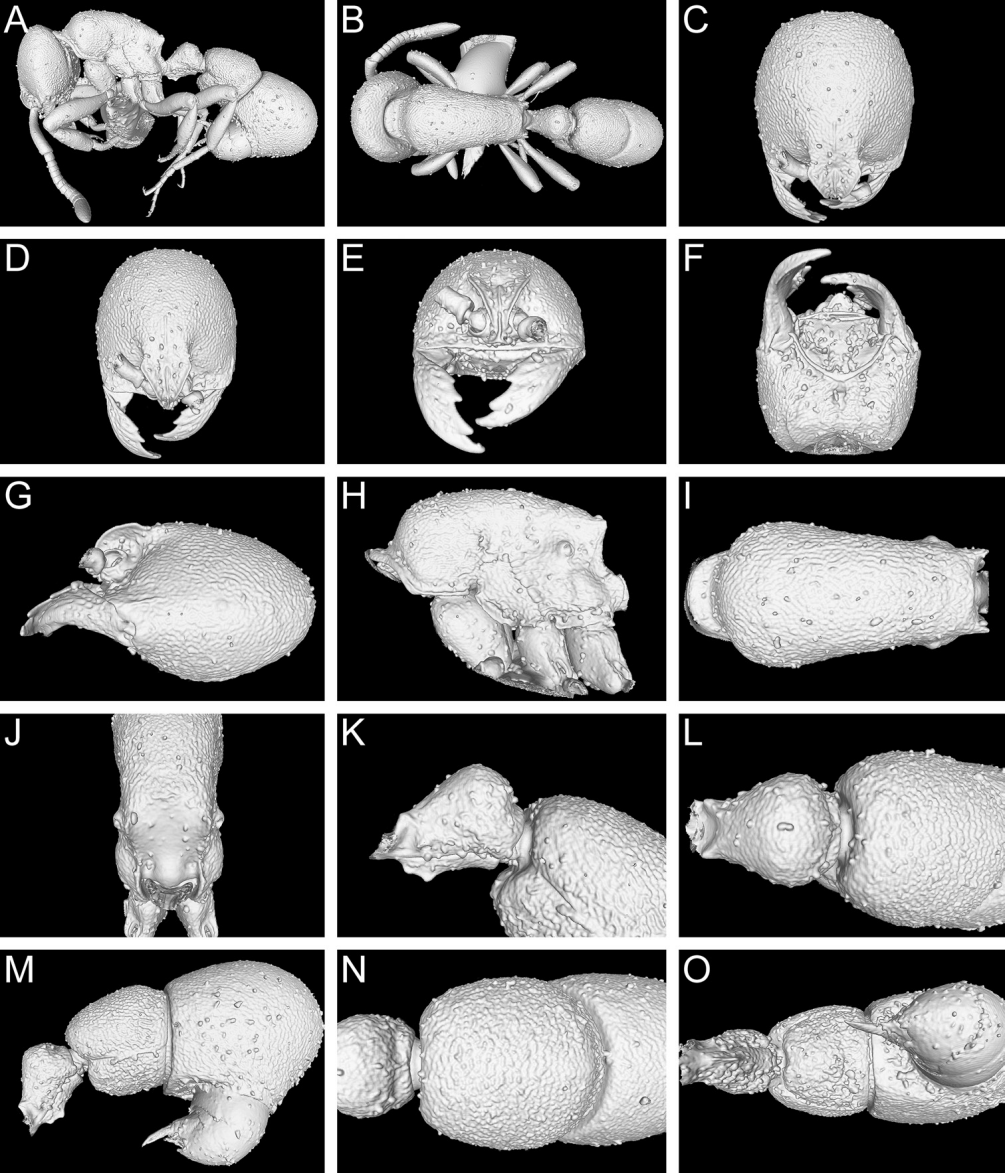
Still images from surface display volume renderings of 3D model of *Proceratium bruelheidei* **sp. n.** holotype worker (CASENT0790023). **A** Body in profile **B** Body in dorsal view **C** Head in dorsal view **D** Head in anterodorsal view **E** Head in anterior view **F** Head in ventral view **G** Head in profile **H** Mesosoma in profile **I** Mesosoma in dorsal view **J** Propodeum in posterodorsal view **K** Abdominal segment II and parts of III in profile **L** Abdominal segment II and parts of III in dorsal view **M** Abdominal segments II–VII in profile **N** Abdominal segment III and parts of II and IV in dorsal view **O** Abdominal segments II–VII in ventral view.

Figs 4B , 6B , 7B , 7D , 8 , 9 , 24

##### Type material.

**Holotype.** Pinned worker from CHINA, Jiangxi Province, near the village Xingangshan, ca. 15 km SE of Wuyuan, 29°7'24"N / 117°54'25"E, 158 m asl, early successional tree plantation of the BEF-China experiment, Winkler leaf litter extraction, 26-IV-2015, leg. Merle Noack, label “MN290” (CASENT0790023), deposited in SWFU.

**Paratypes.** Seven pinned workers in total; one with same data as holotype except label “MN291” (CASENT0790025, in SWFU); one with same data as holotype except 29°7'24"N / 117°54'31"E, 204 m asl, 22-IV-2015, label “MN248” (CASENT0790024, in SWFU); one with same data as holotype except 29°7'33"N / 117°54'41"E, 246 m asl, 30-IV-2015, label “MN309” (CASENT0790029, in SWFU); one with same data as holotype except 29°7'33"N / 117°54'40"E, 239 m asl, 04-V-2015, label “MN371” (CASENT0790030, in SWFU); one with same data as holotype except 29°7'15"N / 117°54'22"E, 122 m asl, 12-V-2015, label “MN479” (CASENT0790028, in CASC); one with same data as holotype except 29°7'37"N / 117°54'25"E, 219 m asl, 20-V-2015, label “MN525” (CASENT0790026, in ZMBH); one from CHINA, Zhejiang Province, Gutianshan National Nature Reserve, ca. 35 km NW of Kaihua, 29°16'37"N / 118°5'26"E, 617 m asl, young secondary subtropical mixed forest, manual sifting of leaf litter, 11-VII-2008, leg. Andreas Schuldt, label “CSP 22” (CASENT0790027, in ZMBH).

**Cybertype.** Volumetric raw data (in DICOM format), 3D rotation video (in .mp4 format, see Suppl. material 3: Video 1), still images of surface volume rendering, and 3D surface (in PLY format) of the physical holotype (CASENT0790023) in addition to montage photos illustrating head in full-face view, profile and dorsal views of the body. The data is deposited at Dryad (Staab et al. 2018, http://dx.doi.org/10.5061/dryad.h6j0g4p) and can be freely accessed as virtual representation of the type. In addition to the cybertype data at Dryad, we also provide a freely accessible 3D surface model of the holotype at Sketchfab (https://skfb.ly/6txMz).

##### Diagnosis.

*Proceratium bruelheidei* differs from the other members of the *P. itoi* clade by the following character combination: relatively large species (TL 3.61–4.00); sides of head straight to very weakly convex, posterior sides only narrowing dorsally, vertex convex; frontal carinae well developed, with large lamellae that extend laterally above the antennal insertions; frontal furrow inconspicuous and of the same color as the surrounding anterior cephalic dorsum; posterodorsal corners of the propodeum broadly angular; propodeal declivity superficially punctured, but shiny; posterior face of petiolar node in profile as steep as anterior face and less than half as long as anterior face; apex of the petiolar node almost as long as broad in dorsal view; subpetiolar process roughly trapezoid and well developed (albeit with variable ventral outline); abdominal segment IV very strongly recurved (IGR 0.24–0.26); in addition to dense pubescence, abundant erect hairs present on scapes and dorsal surface of body, longest of those hairs longer than maximum dorsoventral diameter of metafemur.

##### Worker measurements.

**Holotype.** TL 3.94; EL 0.04; SL 0.50; HL 0.84; HLM 1.09; HW 0.79; WL 1.06; MFeL 0.69; MTiL 0.54; MBaL 0.40; PeL 0.39; PeW 0.32; LT3 0.56; LS4 0.21; LT4 0.84; OI 4; CI 93; SI 59; MFeI 87; MTiI 69; MBaI 51; DPeI 81; IGR 0.25; ASI 150.

**Paratypes (n = 7).** TL 3.61–4.00; EL 0.03–0.04; SL 0.49–0.53; HL 0.79–0.86; HLM 0.96–1.08; HW 0.73–0.79; WL 1.03–1.10; MFeL 0.63–0.74; MTiL 0.54–0.58; MBaL 0.39–0.41; PeL 0.36–0.39; PeW 0.30–0.32; LT3 0.51–0.58; LS4 0.19–0.22; LT4 0.75–0.92; OI 4–5; CI 89–94; SI 60–63; MFeI 86–96; MTiI 69–74; MBaI 50–54; DPeI 82–84; IGR 0.24–0.26; ASI 145–159.

##### Worker description.

In full-face view, head slightly longer than broad (CI 89–94), anterior sides straight to very weakly convex, posterior sides narrowing dorsally, vertex convex. Clypeus reduced and narrow, with a broadly triangular median anterior projection. Frontal carinae relatively short, moderately separated, slightly covering antennal insertions, constantly diverging posteriorly, lateral expansions of anterior part of frontal carinae developed as broad lamellae, raised, conspicuously and broadly extending laterally above antennal insertions; frontal area convex; frontal furrow developed as a raised carina, starting at the clypeal projection and extending over the anterior 2/5 of the cephalic dorsum, with a short gap at the level where the lamellae of frontal carinae are broadest, frontal furrow less conspicuous after the gap. Eyes reduced, minute (OI 4–5), consisting of one to four ommatidia and located on midline of head. Antennae 12-segmented, scapes short (SI 59–63), not reaching posterior head margin and thickening apically. Mandibles elongate and triangular, relatively slender, masticatory margin with four teeth in total, apical tooth long and acute, the other teeth smaller and decreasing in size from second to fourth tooth, gap between second and third tooth larger than between other teeth.

Mesosoma in profile slightly convex and as long as maximum head length including mandibles (WL 1.03–1.10 vs HLM 0.96–1.09). Lower mesopleurae (katepisterna) with well-demarcated sutures, upper mesopleurae (anepisterna) with inconspicuous sutures, no other sutures developed on lateral and dorsal mesosoma; lower mesopleurae weakly inflated posteriorly; posterodorsal corner of propodeum broadly angular, propodeal lobes weakly developed as bluntly rounded lamellae; propodeal declivity almost vertical, slightly inclined anteriorly; in posterodorsal view, sides of propodeum separated from declivity by distinct lamellate margins; in profile view, propodeal spiracle rounded, at mid height, opening of spiracle slightly facing posteriorly. Legs moderately long (MFeL 0.63–0.74, MTiL 0.54–0.58, MBaL 0.39–0.41); all tibiae with a pectinate spur; calcar of strigil without a basal spine; pretarsal claws simple; arolia present.

Petiolar node in profile high, nodiform, with a straight and sloping anterior face, dorsum of node broadly rounded, posterior face as steep as anterior face and relatively short, less than half as long as anterior face; petiole in dorsal view longer than broad, apex of node almost as long as broad; ventral process of petiole well developed, with a roughly trapezoid projection of varying shape and ventral outline (see ‘variation’).

In dorsal view abdominal segment III anteriorly much broader than petiole; its sides convex; abdominal sternite III anteriomedially with a conspicuous depression marked by a thin rim. Constriction between abdominal segments III and IV deep. Abdominal segment IV very large, strongly recurved (IGR 0.24–0.26) and posteriorly rounded, with a lamella on its anterior border around the constriction to abdominal segment III, this lamella thicker ventrally than dorsally; abdominal tergum IV 1.5–1.6× longer than abdominal tergum III (ASI 145–159), remaining abdominal tergites and sternites inconspicuous and projecting anteriorly. Sting large and extended.

Whole body covered with dense mat of short, decumbent to suberect pubescent hairs; additionally, dorsal surfaces of body with abundant significantly longer suberect and erect hairs; such hairs also present on abdominal sterna III + IV, scapes (anterior faces of scapes with many hairs, posterior faces with fewer hairs) and legs (ventral faces of femora and tibiae with many hairs, dorsal faces with fewer hairs), the longest hairs on dorsal surface of body longer than the maximum dorsoventral diameter of metafemur. Mandibles striate; entire body densely punctate; on sides of pronotum punctures aligned in diffuse lines, appearing striate; punctures on antennae, legs, and abdominal segment IV finer than on rest of body; propodeal declivity shiny and at most superficially punctate; abdominal segments V–VII very superficially reticulate and shiny. Body color uniformly orange brown to reddish brown, vertex of head slightly darker, legs, antennal funiculus, and abdominal segments V–VII yellowish brown.

##### Etymology.

The species epithet is a patronym in honor of the German botanist Prof. Helge Bruelheide and his efforts in establishing and promoting the BEF-China project. All specimens of this species were collected on BEF-China field sites.

##### Distribution and ecology.

Most of the type series was collected during a leaf litter ant survey (Noack 2016) in the experimental tree plantations of the BEF-China Main Experiment (Bruelheide et al. 2014). No direct observations of biology and natural history are available. The trees under which the Winkler samples yielding seven of eight type specimens were collected were just six years old and had a mean diameter at breast height of 5.6 ± 2.5 cm (n=7) (Noack 2016). This may indicate that *P. bruelheidei* could prefer early successional forests with a relatively open soil, as the ground from which leaf litter was taken had a mean litter cover of 55 ± 24% (n=7). The single specimen (CASENT0790027) from the Gutianshan National Nature Reserve was likewise collected from an early successional forest stand that was clear-cut less than 20 years prior to the collection of the specimen. However, further sampling will be necessary to draw quantitative conclusions on habitat preferences.

##### Taxonomic notes.

*Proceratium bruelheidei* is most similar to *P. kepingmai*. From the other species of the *P. itoi* clade, *P. bruelheidei* can be separated by using the characters given in the ‘taxonomic notes’ of *P. kepingmai* below. From this species, *P. bruelheidei* differs by the shape of the head in full-face view with straight sides and a convex vertex (sides convex, broadest at level of eyes and vertex almost straight in *P. kepingmai*), the shiny propodeal declivity that is only superficially punctured (densely punctured and mostly opaque in *P. kepingmai*), the inconspicuous frontal furrow that has the same color as the surrounding anterior cephalic dorsum (frontal furrow conspicuous and dark in *P. kepingmai*), the posterior face of petiolar node as steep as the anterior face of the node and less than half as long as the anterior face (posterior face steeper than anterior face and about half as long in *P. kepingmai*), the apex of the petiolar node that is little broader than long (clearly broader than long in *P. kepingmai*), and the more strongly recurved abdominal segment IV (IGR 0.24–0.26) (IGR 0.30–0.32 in *P. kepingmai*). Additionally, *P. bruelheidei* has distinctly more and longer erect hairs protruding from the dense pubescence on the dorsum of the body and the ventral abdomen. While the number of hairs may be a treacherous character as hairs can break during specimen processing, the length of hairs can reliably be quantified. In *P. bruelheidei* the longest erect hairs on the dorsum of the petiole and on abdominal sternum III are longer than the maximum dorsoventral diameter of the metafemur (as long as or shorter than maximum diameter of metafemur in *P. kepingmai*).

##### Variation.

The variation in body size is within the normal limits of other *Proceratium* species and the type specimens of *P. bruelheidei* show, with the notable exception of the subpetiolar process, no observable intraspecific differences. While the process is well developed and roughly trapezoid in all available specimens, its size, exact shape, and ventral outline vary. In the holotype (CASENT0790023) and several paratypes (CASENT0790025, CASENT0790026, CASENT0790029) the subpetiolar process has a distinct notch, so that it almost looks like an upside-down volcano. This notch is absent in other specimens (CASENT0790027, CASENT0790028, CASENT0790030), where the ventral outline of the process is straight. In one specimen (CASENT0790024) the ventral outline is also straight but with a row of minute denticles. It thus appears that this character, which is often used to delimitate *Proceratium* species (e.g. Baroni Urbani and de Andrade 2003, Hita Garcia et al. 2014), may be less suitable for species in the *P. itoi* clade, as also indicated by the variation in the subpetiolar process within the type series of *P. zhaoi* (Xu 2000).

#### Proceratium itoi

Taxon classificationAnimaliaORDOFAMILIA

(Forel, 1918)

**Figure 10.**
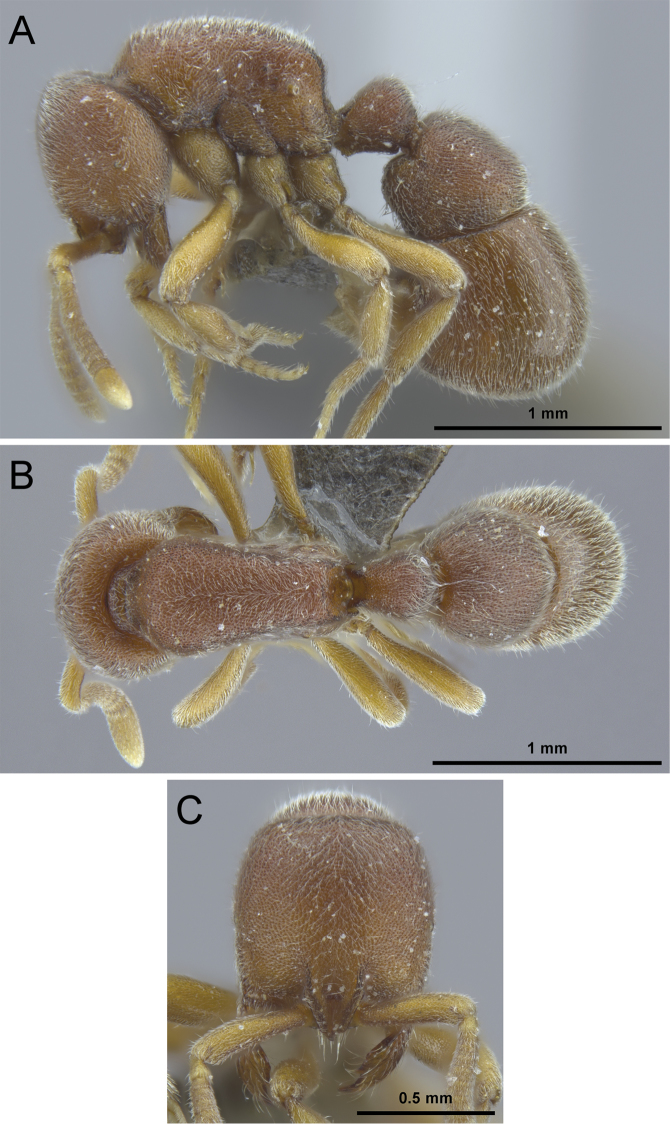
*Proceratium itoi* non-type worker (OKENT0016142). **A** Body in profile **B** Body in dorsal view **C** Head in full-face view.

**Figure 11.**
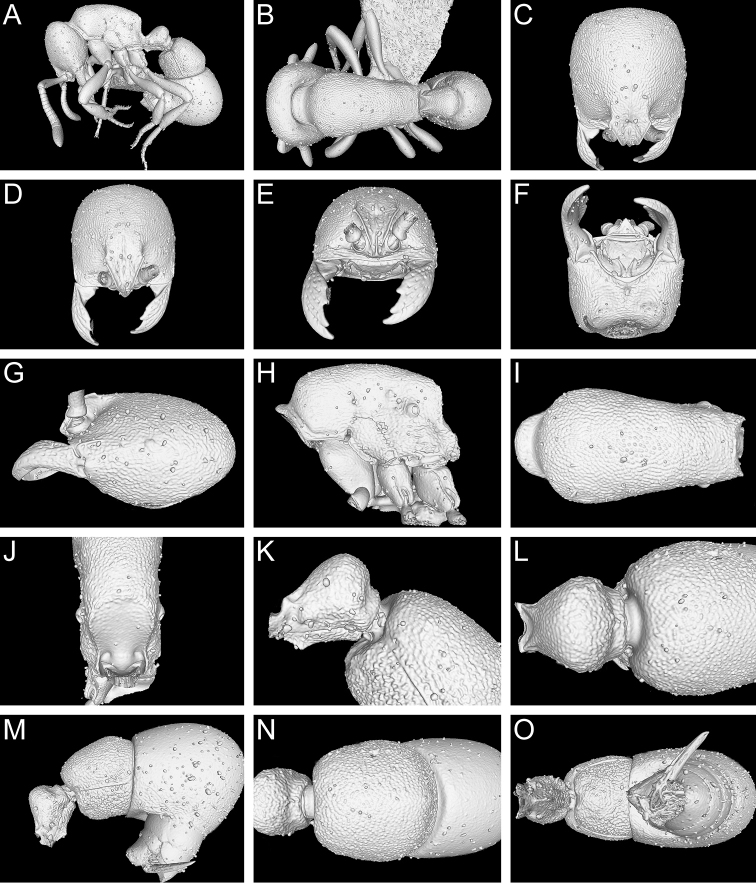
Still images from surface display volume renderings of 3D model of *Proceratium itoi* non-type worker (OKENT0016142). **A** Body in profile **B** Body in dorsal view **C** Head in dorsal view **D** Head in anterodorsal view **E** Head in anterior view **F** Head in ventral view **G** Head in profile **H** Mesosoma in profile **I** Mesosoma in dorsal view **J** Propodeum in posterodorsal view **K** Abdominal segment II and parts of III in profile **L** Abdominal segment II and parts of III in dorsal view **M** Abdominal segments II–VII in profile **N** Abdominal segment III and parts of II and IV in dorsal view **O** Abdominal segments II–VII in ventral view.

Figs 1B , 3B , 3D , 5A , 10 , 11 , 24

Sysphincta itoi Forel, 1918: 717 (w.), Japan
Proceratium itoi – Brown 1958: 247 (see also Baroni Urbani and de Andrade 2003: 267, Onoyama and Yoshimura 2002: 32)
Proceratium itoi – Ogata 1987: 107 (m.), Japan
Proceratium itoi – Onoyama and Yoshimura 2002: 35 (q.), Japan

##### Type material.

**Syntypes.** Three pinned workers from JAPAN, Tokyo, leg. Ito (CASENT0907205, in MHNG) [images examined].

##### Non-type material examined.

JAPAN, Fukuoka, Mt. Tachibana, 25-VII-1984, leg. S. Nomura (OKENT016137; OKENT016138; OKENT016139; OKENT016141; OKENT016142, all in OIST).

##### Virtual dataset.

Volumetric raw data (in DICOM format), 3D rotation video (in .mp4 format, see Suppl. material 4: Video 2), still images of surface volume rendering, and 3D surface (in PLY format) of a non-type specimen (OKENT0016142) in addition to montage photos illustrating head in full-face view, profile and dorsal views of the body. The data is deposited at Dryad (Staab et al. 2018, http://dx.doi.org/10.5061/dryad.h6j0g4p) and can be freely accessed as virtual representation of the species. In addition to the data at Dryad, we also provide a freely accessible 3D surface model at Sketchfab (https://skfb.ly/6txMM).

##### Diagnosis.

*Proceratium itoi* differs from the other members of the *P. itoi* clade by the following character combination: medium-sized species (TL 3.46–3.82); sides of head very weakly convex, almost straight, broadest at level of eyes and gently narrowing anteriorly and posteriorly, vertex weakly convex, almost straight; frontal carinae well developed, with large lamellae that extend laterally above the antennal insertions; frontal furrow inconspicuous; posterodorsal corners of propodeum rounded, propodeal declivity superficially punctured (more so dorsally) but largely shiny; posterior face of petiolar node in profile steeper as anterior face; petiole almost as broad as long (DPeI 86–93), apex of petiolar node broader than long in dorsal view; subpetiolar process developed and triangular (but may be small); in addition to dense pubescence abundant erect hairs present on scapes and dorsal surface of body, longest of those hairs shorter than maximum dorsoventral diameter of metafemur.

##### Distribution and ecology.

This species is widely distributed, occurring from Japan (except Hokkaido) and South Korea to Vietnam. It has been recorded from Taiwan and the Chinese provinces Zhejiang and Hunan. Thus, we expect that it will be collected from the geographically intermediate provinces in the future. No direct biological observations from China are available, but the Japanese populations are comparatively well studied (Onoyama and Yoshimura 2002). Nests are found in the soil or rotting wood of various deciduous or evergreen forest types and workers forage hypogeic or in leaf litter. Mature colonies have 100–200 workers and densities can reach 0.3 colonies per m² (Masuko 2010). Larval hemolymph feeding has been observed (Masuko 1986).

##### Taxonomic notes.

*Proceratium itoi* is a typical member of its clade of intermediate size (WL 0.96–1.04) and is similar to most other species in body proportions and indices. *Proceratium itoi* can be separated from *P. williamsi* and *P. zhaoi* by the presence of erect hairs on the dorsal body surface (absent in *P. williamsi* and *P. zhaoi*); from *P. longmenense* by the presence of erect hairs on the scape (absent in *P. longmenense*) and by the frontal carinae separated at their anteriormost level (touching each other at their anteriormost level in *P. longmenense*). In *P. itoi* the posterodorsal corners of propodeum are rounded and this character distinguishes this species from *P. bruelheidei* and *P. kepingmai* (posterodorsal corners of the propodeum angular), which are also larger species (WL 1.03–1.10 and 1.14–1.24). The rounded posterodorsal corners of propodeum are shared between *P. malesianum* and *P. itoi*, but *P. malesianum* is a smaller species (WL 0.71–0.90) with a broadly rounded vertex (weakly convex, almost straight in *P. itoi*) and a broadly rounded petiolar node in profile (posterior face of petiolar node in profile steeper than anterior face in *P. itoi*).

#### Proceratium kepingmai

Taxon classificationAnimaliaORDOFAMILIA

Staab, Xu & Hita Garcia sp. n.

http://zoobank.org/0233F3AD-8F60-4CBB-B64B-B0A48857CBFF

**Figure 12.**
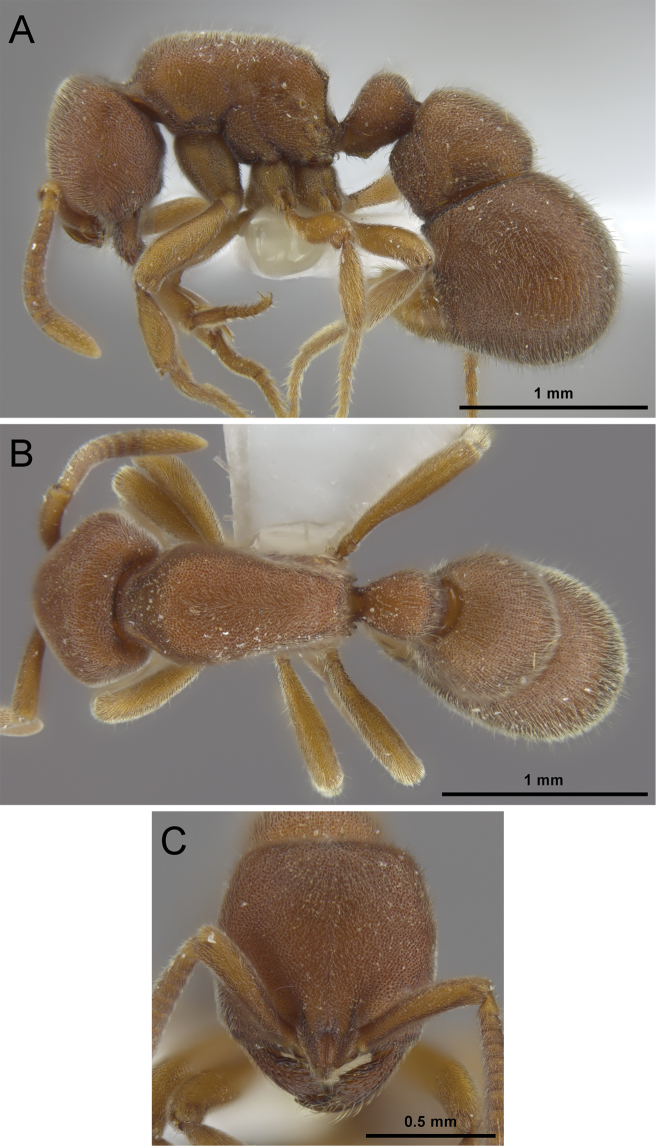
*Proceratium kepingmai* **sp. n.** holotype worker (CASENT0790031). **A** Body in profile **B** Body in dorsal view **C** Head in full-face view.

**Figure 13.**
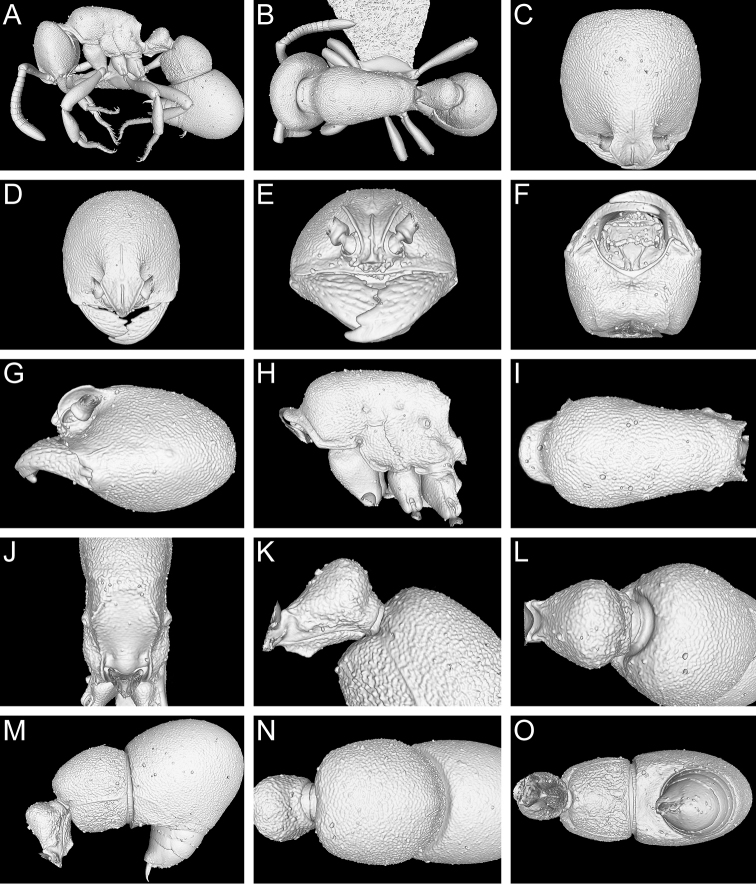
Still images from surface display volume renderings of 3D model of *Proceratium kepingmai* **sp. n.** holotype worker (CASENT0790031). **A** Body in profile **B** Body in dorsal view **C** Head in dorsal view **D** Head in anterodorsal view **E** Head in anterior view **F** Head in ventral view **G** Head in profile **H** Mesosoma in profile **I** Mesosoma in dorsal view **J** Propodeum in posterodorsal view **K** Abdominal segment II and parts of III in profile **L** Abdominal segment II and parts of III in dorsal view **M** Abdominal segments II–VII in profile **N** Abdominal segment III and parts of II and IV in dorsal view **O** Abdominal segments II–VII in ventral view.

Figs 5B , 7A , 7C , 12 , 13 , 24

##### Type material.

**Holotype.** Pinned worker from CHINA, Jiangxi Province, near the village Xingangshan, ca. 15 km SE of Wuyuan, 29°7'28"N / 117°54'40"E, 270 m asl, secondary subtropical mixed forest, Winkler leaf litter extraction, 26-III-2015, leg. Michael Staab, label “MS1836” (CASENT0790031), deposited in SWFU.

**Paratype.** Pinned worker from CHINA, Zhejiang Province, Gutianshan National Nature Reserve, ca. 30 km NW of Kaihua, 29°14'50"N / 118°8'10"E, 665 m asl, secondary subtropical mixed forest, Winkler leaf litter extraction, 27-IV-2015, leg. Merle Noack, label “MS1859” (CASENT0790032), deposited in ZMBH.

**Cybertype.** Volumetric raw data (in DICOM format), 3D rotation video (in .mp4 format, see Suppl. material 5: Video 3), still images of surface volume rendering, and 3D surface (in PLY format) of the physical holotype (CASENT0790031) in addition to montage photos illustrating head in full-face view, profile and dorsal views of the body. The data is deposited at Dryad (Staab et al. 2018, http://dx.doi.org/10.5061/dryad.h6j0g4p) and can be freely accessed as virtual representation of the type. In addition to the cybertype data at Dryad, we also provide a freely accessible 3D surface model of the holotype at Sketchfab (https://skfb.ly/6txMy).

##### Diagnosis.

*Proceratium kepingmai* differs from the other members of the *P. itoi* clade by the following character combination: large species (TL 4.39–4.54); sides of head weakly convex, broadest at level of eyes and gently narrowing anteriorly and stronger posteriorly; vertex almost straight; very reduced eyes (OI 2–3) consisting of a single minute ommatidium; frontal carinae well developed, with large lamellae that extend laterally above the antennal insertions; frontal furrow darker than the surrounding anterior cephalic dorsum; posterodorsal corners of the propodeum broadly angular; propodeal declivity densely punctured, mostly opaque; posterior face of petiolar node in profile steeper than anterior face and about half as long as anterior face; apex of petiolar node distinctly broader than long in dorsal view; in addition to dense pubescence, erect hairs present on scapes and dorsal surface of body, longest of those hairs at most as long as the maximum dorsoventral diameter of metafemur.

##### Worker measurements.

**Holotype.** TL 4.39; EL 0.02; SL 0.57; HL 0.92; HLM 1.08; HW 0.86; WL 1.14; MFeL 0.71; MTiL 0.60; MBaL 0.44; PeL 0.45; PeW 0.36; LT3 0.64; LS4 0.32; LT4 1.08; OI 2; CI 92; SI 60; MFeI 83; MTiI 70; MBaI 51; DPeI 80; IGR 0.30; ASI 169.

**Paratype.** TL 4.54; EL 0.03; SL 0.59; HL 0.98; HLM 1.14; HW 0.90; WL 1.24; MFeL 0.79; MTiL 0.65; MBaL 0.48; PeL 0.46; PeW 0.37; LT3 0.65; LS4 0.34; LT4 1.05; OI 3; CI 93; SI 62; MFeI 88; MTiI 72; MBaI 53; DPeI 80; IGR 0.33; ASI 161.

##### Worker description.

In full-face view, head slightly longer than broad (CI 92–93), sides weakly convex, broadest at the eye level and gently narrowing anteriorly and (stronger) posteriorly, vertex weakly convex, almost straight. Clypeus reduced and narrow, with a broadly triangular median anterior projection. Frontal carinae relatively short, moderately separated, slightly covering antennal insertions, constantly diverging posteriorly, lateral expansions of anterior part of frontal carinae developed as broad lamellae, raised, conspicuously and broadly extending laterally above antennal insertions; frontal area convex; frontal furrow well developed as a raised carina, starting at the clypeal projection and extending over the anterior 2/5 of the cephalic dorsum, with a short gap at the level where the lamellae of frontal carinae are broadest. Eyes reduced, minute (OI 2–3), consisting of a single ommatidium and located on midline of head. Antennae 12-segmented, scapes short (SI 60–62), not reaching posterior head margin and thickening apically. Mandibles elongate and triangular, masticatory margin with four teeth in total, apical tooth long and acute, the other teeth smaller and decreasing in size from second to fourth tooth, gap between second and third tooth larger than between other teeth.

Mesosoma in profile slightly convex and slightly longer than maximum head length including mandibles (WL 1.14–1.24 vs. HLM 1.08–1.14). Lower mesopleurae (katepisterna) with well-demarcated sutures, upper mesopleurae (anepisterna) with inconspicuous sutures, no other sutures developed on lateral and dorsal mesosoma; lower mesopleurae weakly inflated posteriorly; posterodorsal corner of propodeum broadly angular, propodeal lobes weakly developed as bluntly rounded lamellae; propodeal declivity almost vertical, slightly inclined anteriorly; in posterodorsal view sides of propodeum separated from declivity by distinct lamellate margins; in profile propodeal spiracle rounded, at mid height, opening of spiracle slightly facing posteriorly. Legs moderately long; all tibiae with a pectinate spur; calcar of strigil without a basal spine; pretarsal claws simple; arolia present.

Petiolar node in profile high, nodiform, with a straight and sloping anterior face, dorsum of node broadly rounded, posterior face half as long and steeper than anterior face; petiole in dorsal view longer than broad but apex of node clearly broader than long; ventral process moderately developed on anterior petiole, with a relatively indistinct rectangular projection.

In dorsal view abdominal segment III anteriorly much broader than petiole; its sides convex; abdominal sternite III anteriomedially with a conspicuous depression marked by a thin rim. Constriction between abdominal segments III and IV deep. Abdominal segment IV very large, recurved (IGR 0.30–0.33) and posteriorly strongly rounded, with a lamella on its anterior border around the constriction to abdominal segment III, this lamella thicker ventrally than dorsally; abdominal tergum IV 1.6–1.7× longer than abdominal tergum III (ASI 161–169); remaining abdominal tergites and sternites inconspicuous and projecting anteriorly. Sting large and extended.

Whole body covered with dense mat of short, decumbent to suberect pubescent hairs; additionally, the dorsal surfaces of body interspersed with significantly longer suberect and erect hairs, such hairs also present on abdominal sterna III + IV, scapes (anterior faces of scapes with many hairs, posterior faces with single hairs), and legs (ventral faces of femora and tibiae with many hairs, dorsal faces with single hairs); the longest hairs on dorsal surface of body at most as long as the maximum dorsoventral diameter of metafemur. Mandibles striate; entire body including propodeal declivity densely punctate; on sides of pronotum punctures aligned in diffuse lines, appearing striate; punctures on antennae, legs, and abdominal segment IV finer than on rest of body, abdominal segments V–VII very superficially punctured and shiny. Body color uniformly orange brown to reddish brown, vertex of head slightly darker, frontal furrow conspicuously darker than surrounding cephalic dorsum, legs, antennal funiculus, and abdominal segments V–VII yellowish brown.

##### Etymology.

The species epithet is a patronym in honor of the Chinese botanist Prof. Keping Ma and his efforts in establishing the BEF-China project and promoting biodiversity research and nature conservation in China. All specimens of this species were collected in old-growth subtropical forest, an ecosystem Prof. Ma has investigated in detail.

##### Distribution and ecology.

Both specimens were collected in secondary mixed evergreen broadleaved forest of relatively advanced age, as indicated by the presence of large trees. The paratype was collected within the Gutianshan National Nature Reserve (Yu et al. 2001, Bruelheide et al. 2011, Staab 2014), one of the larger remaining fragments of subtropical broadleaved forest in southeast China. The forest at this locality (the type locality is a similar but much smaller forest fragment) is on sloped land and rich in plant species; more than 250 woody species have been recorded on about 8000 ha. Approximately 50% of the woody species are deciduous but the tree layer is dominated by evergreen species including *Castanopsis eyrei* (Fagaceae) (Champ. ex Benth.) Tutch., *Cyclobalanopsis glauca* (Fagaceae) (Thunb.) Oerst., *Machilus thunbergii* (Lauraceae) Sieb. et Zucc., and *Schima superba* (Theaceae) Gardn. et Champ. No direct observations of biology and natural history are available for *P. kepingmai*.

##### Taxonomic notes.

*Proceratium kepingmai* is the largest (WL 1.14–1.24) member of the *P. itoi* clade and has, even for eye-bearing *Proceratium*, very minute eyes (OI 2–3). From each of the species in the clade with very similar body proportions (particularly indices) that also have erect hairs on the dorsal surface of the body (*P. itoi*, *P. longmenense*, *P. malesianum*, *P. bruelheidei*; no erect hairs, only dense pubescence in *P. williamsi*, *P. zhaoi*) it can safely be separated by one or more characters. In *P. kepingmai* the posterodorsal corner of the propodeum is angular (rounded in *P. itoi* and *P. malesianum*), which is also the case for *P. longmenense* and *P. bruelheidei*. However, *P. longmenense* lacks erect hairs on the scape (at least some erect hairs present in *P. kepingmai* and *P. bruelheidei*), has a relatively narrower head (CI 85) with longer scapes (SI 68) (CI 92–93 and SI 60–62 in *P. kepingmai*), and frontal carinae that touch each other at their anteriormost level (clearly separated in *P. kepingmai* and *P. bruelheidei*). With *P. bruelheidei*, the most similar species, *P. kepingmai* also shares the broad frontal carinae that have large lamellae and are conspicuously extended laterally above the antennal insertions (not extended and narrower in *P. longmenense*). In contrast, *P. kepingmai* differs from *P. bruelheidei* by the shape of the head in full-face view that has convex sides, which are broadest at the level of the eyes and narrow weakly anteriorly and more strongly posteriorly towards to almost straight vertex (sides straight, not narrowing anteriorly and vertex convex in *P. bruelheidei*), the densely punctured and mostly opaque propodeal declivity (sparsely and superficially punctured and very shiny in *P. bruelheidei*), the conspicuous frontal furrow that is darker than the rest of the surrounding anterior cephalic dorsum (inconspicuous and of same color in *P. bruelheidei*), the posterior face of petiolar node in profile steeper than the anterior face of the node and about half as long as the anterior face (posterior face as steep as anterior face and less than half as long in *P. bruelheidei*), the apex of the petiolar node that is clearly broader than long in dorsal view (less broad than long in *P. bruelheidei*), and relatively fewer and shorter erect hairs (see *P. bruelheidei* for details).

##### Variation.

Apart from the small difference in body size (WL 1.14 vs. 1.24) there is no observable variation between the two specimens.

#### Proceratium longmenense

Taxon classificationAnimaliaORDOFAMILIA

Xu, 2006

Figs 6A , 14 , 25

Proceratium longmenense Xu, 2006: 154 (w.), China

##### Type material.

**Holotype.** Pinned worker from CHINA, Yunnan Province, Kunming City, Xishan Mountain Forest Park, Longmen, subtropical evergreen broadleaf forest, 2050 m asl, 5-V-2001, leg. Zhenghui Xu, No. A00514 (in SWFU) [examined].

##### Diagnosis.

*Proceratium longmenense* differs from the other members of the *P. itoi* clade by the following character combination: medium-sized species (TL 3.2); sides of head and vertex weakly convex, almost straight; head (CI 85) and scapes (SI 68) relatively long; frontal carinae developed, their lateral lamellae relatively narrow, touching each other at their anteriormost level, not conspicuously broader above antennal insertions; posterodorsal corners of the propodeum broadly angular; posterior face of petiolar node in profile shorter and steeper than anterior face; petiole almost as broad as long (DPeI 91); subpetiolar process developed, roughly trapezoid; in addition to dense pubescence erect hairs present on dorsal surface of body, but only sparsely on head, scapes without erect hairs.

**Figure 14.**
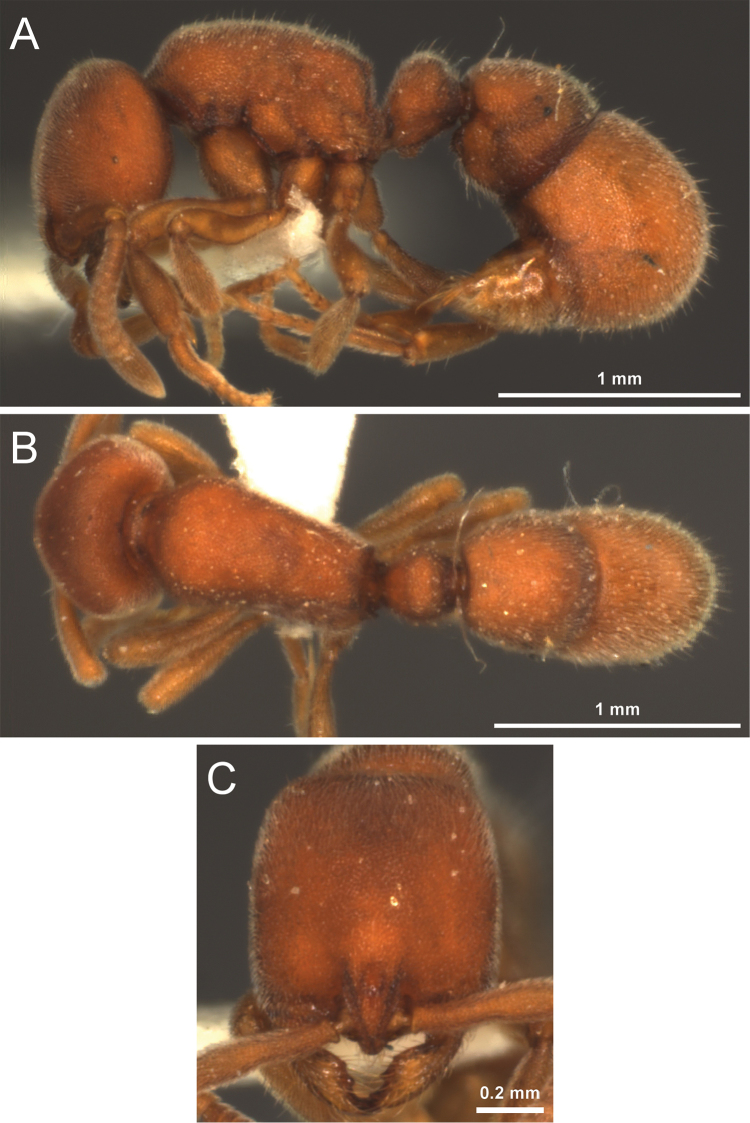
*Proceratium longmenense* holotype worker. **A** Body in profile **B** Body in dorsal view **C** Head in full-face view.

##### Distribution and ecology.

This species is only known from the holotype that was collected in subtropical evergreen broadleaved forest at 2050 m asl. No direct observations of biology and natural history are available for *P. longmenense*.

##### Taxonomic notes.

The unique hair patterns separate *P. longmenense* from the other species of the *P. itoi* clade. *Proceratium williamsi* and *P. zhaoi* have no erect hairs that protrude from the dense pubescence on the dorsal surface of body (hairs present in *P. longmenense*, but relatively sparsely, especially on head). All other species (*P. bruelheidei*, *P. itoi*, *P. kepingmai*, *P. malesianum*) have also such hairs on the scapes (absent on scapes in *P. longmenense*). In addition to hairs, which may be worn down in old specimens, *P. longmenense* is unique by the relatively long scapes (SI 68) combined with the relatively narrow head (CI 85). Among the other Chinese *P. itoi* clade species, it differs furthermore from *P. zhaoi* in size (WL 0.97; WL<80 in *P. zhaoi*), from *P. itoi* by the shape of the posterodorsal corners of the propodeum (broadly angular; rounded in *P. itoi*), and from *P. bruelheidei*, *P. itoi*, and *P. kepingmai* by the lamellae of the frontal carinae (touching each other at their anteriormost level; separated in the other three species).

#### Proceratium zhaoi

Taxon classificationAnimaliaORDOFAMILIA

Xu, 2000

**Figure 15.**
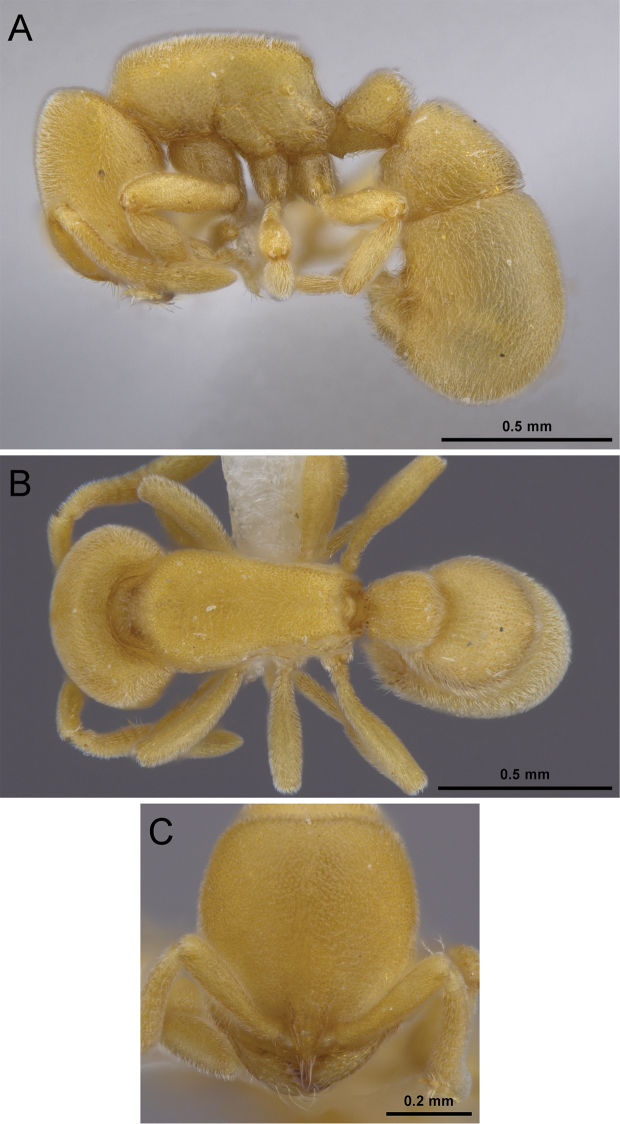
*Proceratium zhaoi* paratype worker (CASENT0790671). **A** Body in profile **B** Body in dorsal view **C** Head in full-face view.

**Figure 16.**
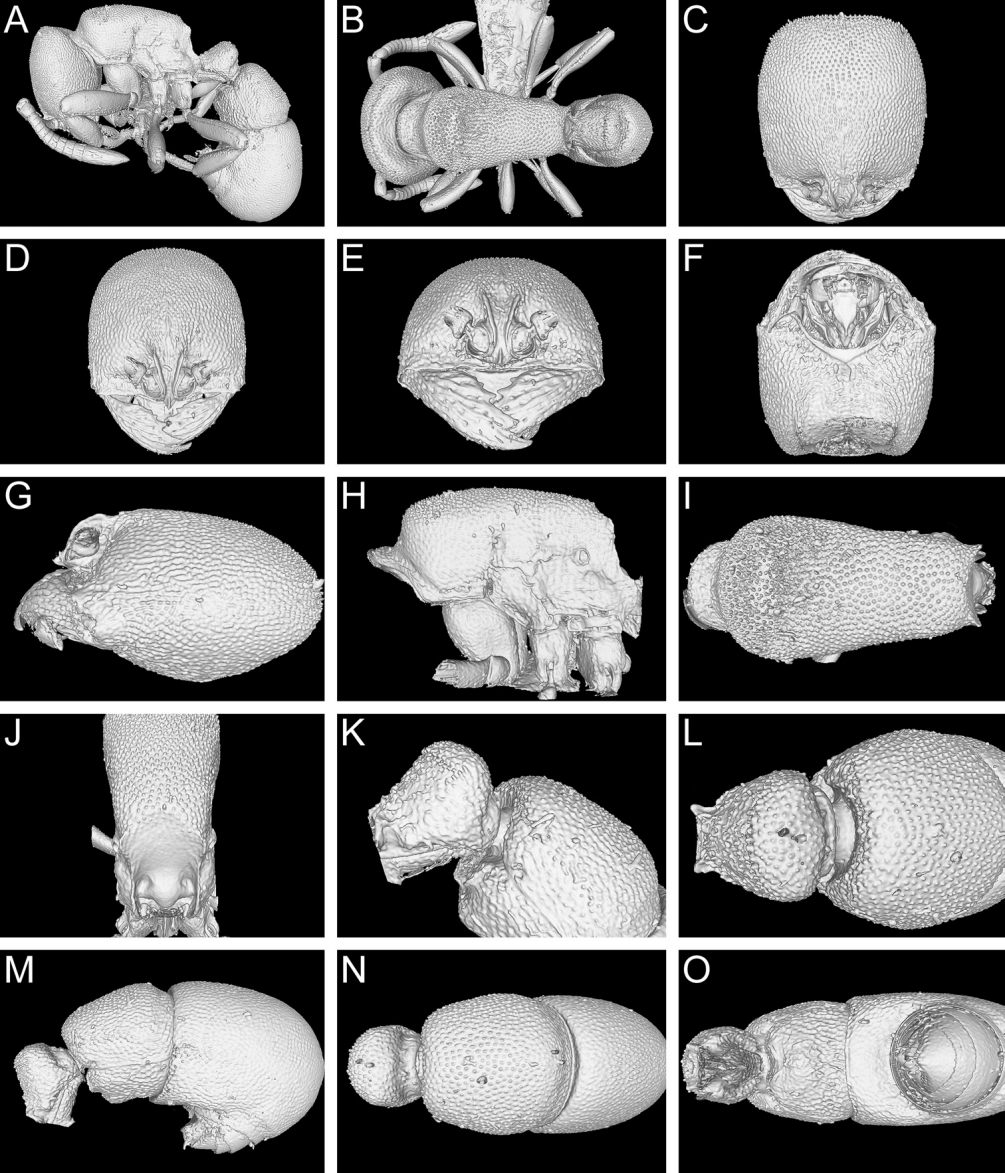
Still images from surface display volume renderings of 3D model of *Proceratium zhaoi* paratype worker (CASENT0790671). **A** Body in profile **B** Body in dorsal view **C** Head in dorsal view **D** Head in anterodorsal view **E** Head in anterior view **F** Head in ventral view **G** Head in profile **H** Mesosoma in profile **I** Mesosoma in dorsal view **J** Propodeum in posterodorsal view **K** Abdominal segment II and parts of III in profile **L** Abdominal segment II and parts of III in dorsal view **M** Abdominal segments II–VII in profile **N** Abdominal segment III and parts of II and IV in dorsal view **O** Abdominal segments II–VII in ventral view.

**Figure 17.**
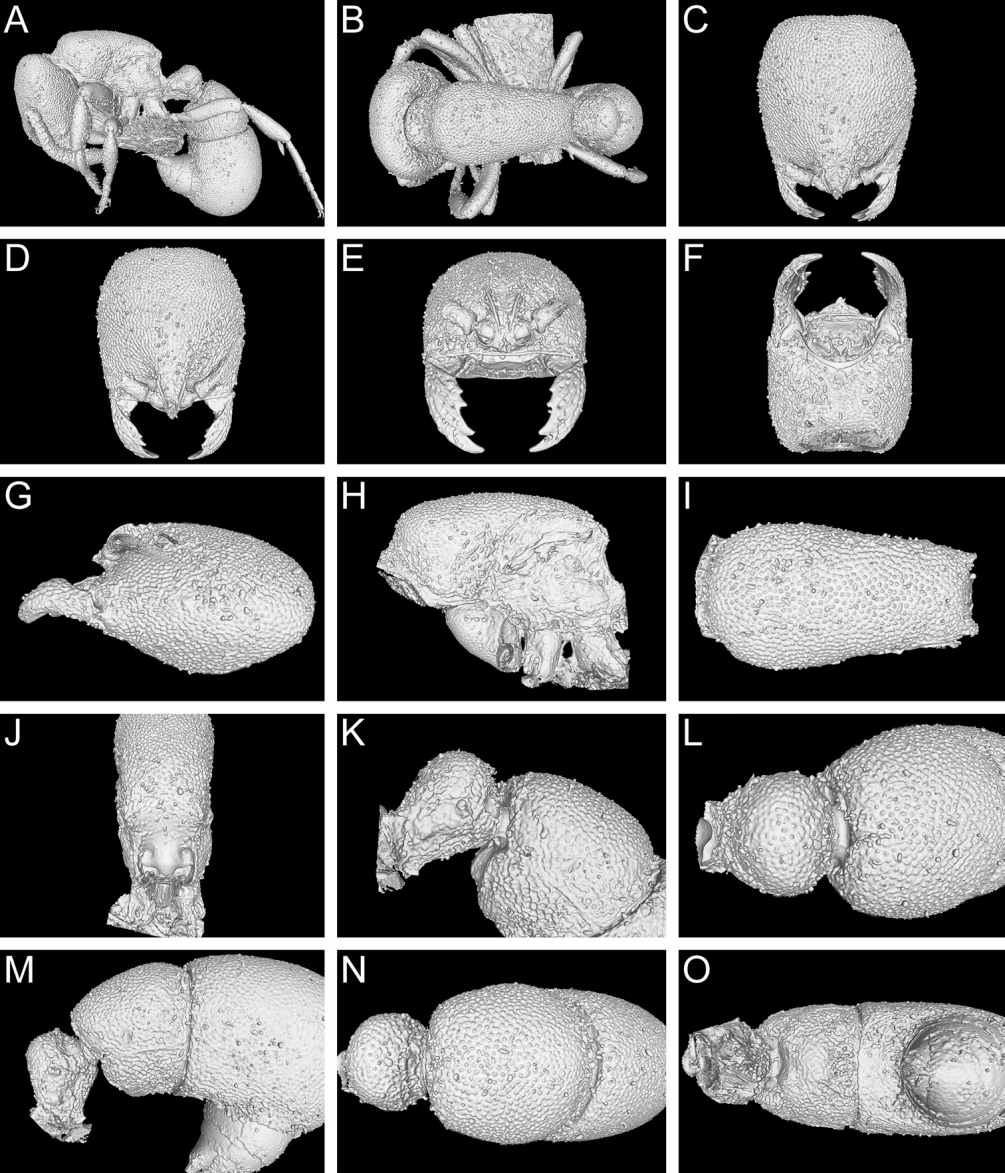
Still images from surface display volume renderings of 3D model of *Proceratium zhaoi* paratype worker of *P. nujiangense* (CASENT0790672). **A** Body in profile **B** Body in dorsal view **C** Head in dorsal view **D** Head in anterodorsal view **E** Head in anterior view **F** Head in ventral view **G** Head in profile **H** Mesosoma in profile **I** Mesosoma in dorsal view **J** Propodeum in posterodorsal view **K** Abdominal segment II and parts of III in profile **L** Abdominal segment II and parts of III in dorsal view **M** Abdominal segments II–VII in profile **N** Abdominal segment III and parts of II and IV in dorsal view **O** Abdominal segments II–VII in ventral view.

Figs 4A , 15 , 16 , 17 , 25

Proceratium zhaoi Xu, 2000: 435 (w.q.), China
Proceratium nujiangense Xu, 2006: 153 (w.q.), China, **syn. n.**

##### Type material.

**Of *P. zhaoi*: Holotype.** Pinned worker from CHINA, Yunnan Province, Menghai County, Meng’a Town, Papo Village, 1280 m asl, deciduous broadleaved forest, soil sample, 10-IX-1997, leg. Zheng-Hui Xu, No. A97-2338 (in SWFU) [examined].

**Paratypes.** Six pinned workers and 24 alate females; one worker with same data as holotype; all other paratypes with same data as holotype but No. A97-2380 (CASENT0235334 in CASC; CASENT0790671 and all other paratypes in SWFU) [all examined].

**Of *P. nujiangense*: Holotype.** Pinned worker from CHINA, Yunnan Province, Baoshan City, Lujiang Town, Bawan, 1500 m asl, *Pinus yunnanensis* forest on east slope of Nujiang River Valley, 11-VIII-1998, leg. Qizhen Long, label “A98-1964” (in SWFU) [examined].

**Paratypes.** Seven pinned workers and 10 queens with same data as holotype but No. A98-1995, No. A98-1997, No. A98-2010, No. A98-2016, No. A98-2029 (CASENT0790672 and all other paratypes in SWFU) [all examined].

##### Virtual dataset.

Volumetric raw data (in DICOM format), 3D rotation videos (in .mp4 format, see Suppl. material 6: Video 4 for *P. zhaoi* and Suppl. material 7: Video 5 for *P. nujiangense*), still images of surface volume rendering, and 3D surfaces (in PLY format) of a paratype of *P. zhaoi* (CASENT07900671) and a paratype of *P. nujiangense* (CASENT0790672) in addition to montage photos illustrating head in full-face view, profile and dorsal views of the body. The data is deposited at Dryad (Staab et al. 2018, http://dx.doi.org/10.5061/dryad.h6j0g4p) and can be freely accessed as virtual representations of the species. In addition to the data at Dryad, we also provide freely accessible 3D surface models at Sketchfab (https://skfb.ly/6txOT and https://skfb.ly/6txOL).

##### Diagnosis.

*Proceratium zhaoi* differs from the other members of the *P. itoi* clade by the following character combination: small species (TL 2.0–2.8, WL 0.66–0.80; measurements and indices use data from the original descriptions); of head weakly convex, broadest at level of eyes and gently narrowing anteriorly and posteriorly, posterior head margin weakly concave to almost straight; frontal carinae developed, their lateral lamellae relatively narrow, not extending over antennal insertions; posterodorsal corners of propodeum bluntly angled; posterior face of petiolar node, in profile, shorter and steeper than anterior face, dorsum of node broadly rounded, petiole as long as broad or broader than long (DPeI 98–110), subpetiolar process developed, relatively variable, varying in size and shape (from rectangular to triangular to acutely toothed); only dense pubescence, no erect hairs on dorsum of body, head, and scapes.

##### Distribution and ecology.

This species is only known from two locations at mid elevation in forests of southern and western Yunnan Province. The original description reported 45 workers in the type colony (Xu 2000) and no other data on natural history have been published. However, the relatively short legs suggest a purely hypogeic life style, which conforms to the fact that specimens were extracted from soil samples.

##### Taxonomic notes.

Even though at the beginning of this study we treated *P. nujiangense* and *P. zhaoi* as distinct species, thorough examinations combining traditional microscopy with micro-CT scans proved that there are no morphological characters separating them. The virtual comparisons of type specimens of both taxa showed that there are no morphological differences, a fact that is not easy to observe by comparing physical specimens. The types are hairy, dirty, and mounted in ways that hide most important characters, as it is typical for most *Proceratium* specimens. Furthermore, the main character used by Xu (2006) to separate the species was the subpetiolar process, which has been used for species diagnostics in previous studies (Baroni Urbani and de Andrade 2003, Hita Garcia et al. 2014, 2015). However, these works either had very little material for the assessment of intraspecific variation and/or treated different clades of *Proceratium*. Our study shows that the subpetiolar process is extremely variable within the *P. itoi* clade and refrain from using it for species delimitations. As a matter of fact, the variation of the subpetiolar process was already noted in the description of *P. zhaoi* (Xu 2000). Reexamination of all type specimens of both species also revealed a comparatively high degree of variation and overlap in the form of the posterodorsal corner of the propodeum and the width of the propodeal node. In addition, the morphometric ranges of *P. nujiangense* and *P. zhaoi* overlap and form a continuum, and there are no significant differences in proportions since all indices are identical. Considering these similarities in light of the newly available images and micro-CT data, we propose treating *P. nujiangense* as a junior synonym of *P. zhaoi*.

This species was not mentioned in the revision of Baroni Urbani and de Andrade (2003), potentially because the authors were not aware of its description shortly before the completion of their monograph. Despite some size variation (TL 2.0–2.8), the relative body proportions of *P. zhaoi* are constant (CI 84–90, SI 61–66). *Proceratium zhaoi* is the smallest (WL 0.66–0.80) member of the *P. itoi* clade. It can be distinguished from all other *P. itoi* clade species (except for *P. williamsi*) by the absence of erect hairs that protrude through the dense pubescence on the dorsal body surface. *Proceratium williamsi* also lacks hairs on the dorsal body surface, but is larger (WL 0.80–0.92), has stronger developed frontal carinae and relatively more slender and longer legs. The relatively weakly developed frontal carinae and the short legs (MFeI <80, MTiI <65, MBaI <40) make *P. zhaoi* also unique among the Chinese *P. itoi* clade species.

#### Proceratium silaceum

Taxon classificationAnimaliaORDOFAMILIA

clade

##### Definition.

Workers of this clade can be distinguished by a moderately squamiform petiolar node that narrows only little from base to apex (extremely squamiform in the Fiji archipelago, Hita Garcia et al. 2015) and by an almost straight to weakly concave anterior clypeal margin (definition follows Baroni Urbani and de Andrade 2003).

##### Comments.

The *P. silaceum* clade sensu Baroni Urbani and de Andrade (2003) is, with more than 30 species, the most speciose and widespread clade within the genus. Numerous species occur in, respectively, Borneo and Australia. Species of this clade have been reported from all continents and several have reached oceanic islands. From China and east Asia only two species, *P. japonicum* and *P. longigaster*, are known.

#### Proceratium japonicum

Taxon classificationAnimaliaORDOFAMILIA

Santschi, 1937

**Figure 18.**
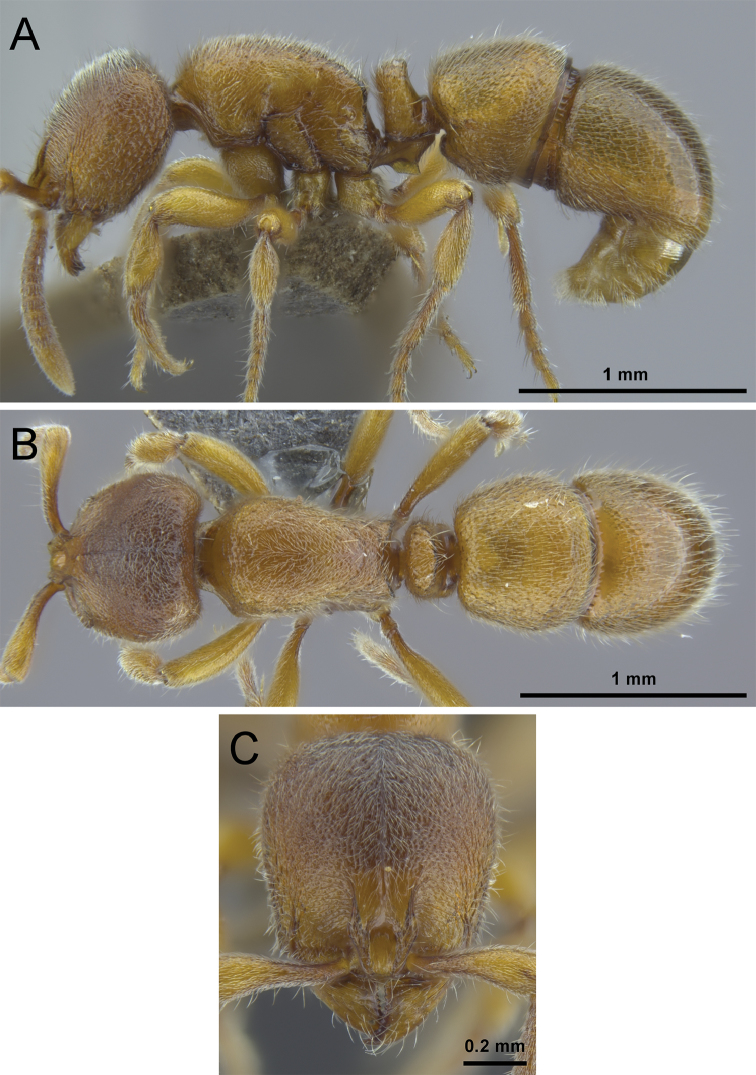
*Proceratium japonicum* non-type worker (CASENT0790834). **A** Body in profile **B** Body in dorsal view **C** Head in full-face view.

**Figure 19.**
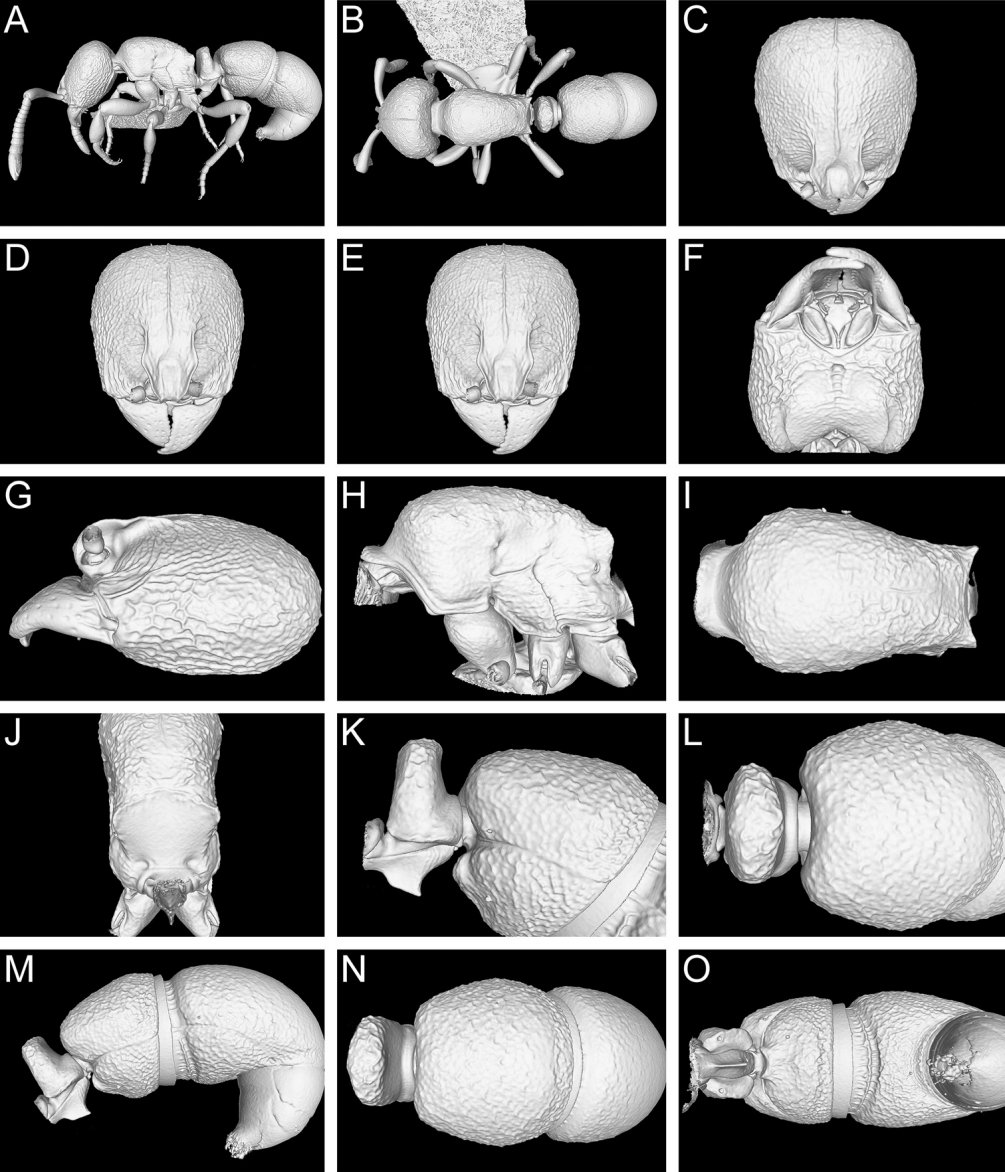
Still images from surface display volume renderings of 3D model of *Proceratium japonicum* non-type worker (CASENT0790834). **A** Body in profile **B** Body in dorsal view **C** Head in dorsal view **D** Head in anterodorsal view **E** Head in anterior view **F** Head in ventral view **G** Head in profile **H** Mesosoma in profile **I** Mesosoma in dorsal view **J** Propodeum in posterodorsal view **K** Abdominal segment II and parts of III in profile **L** Abdominal segment II and parts of III in dorsal view **M** Abdominal segments II–VII in profile **N** Abdominal segment III and parts of II and IV in dorsal view **O** Abdominal segments II–VII in ventral view.

Figs 1A , 2B , 18 , 19 , 24

Proceratium japonicum Santschi, 1937: 362 (w.), Japan (see also Baroni Urbani and de Andrade 2003: 368, Onoyama and Yoshimura 2002: 38)
Proceratium formosicola Terayama, 1985: 406 (w.q.), Taiwan (junior synonym, see Onoyama 1991: 695)
Proceratium japonicum – Onoyama and Yoshimura 2002: 35 (q.m.), Japan

##### Type material.

**Of *P. japonicum*: Syntypes.** Three pinned workers from JAPAN, Honshu, Oshima, Iya, Honshiu, 10.VI.28, leg. K. Sato (CASENT0915312, in NHMB) [images examined].

**Of *P. formosicola*: Holotype.** TAIWAN, Nantou Hsien, Lushan, ca. 100 m asl, 15-VIII.1980, leg. M. Terayama. (in NIAES) [not examined].

**Paratypes.** Two pinned workers and one queen with same data as holotype; one pinned worker from TAIWAN, Nantou Hsien, Puli, 4-VIII.1981, leg. M. Terayama (TARI) [not examined].

##### Non-type material examined.

JAPAN: Okinawa, Ishigaki Island, Mt. Omoto, 1-IV-1975, leg. M. Tanaka (CASENT0281854, in BMNH); JAPAN, Okinawa, Irimote Island, Shirahama, 6-V-2000, leg. M. Yoshimura (OKENT0019995; OKENT0019996, both in OIST); JAPAN, Kanagawa, Odawara, Minazurimisaki, 27-VII-2000, leg. M. Yoshimura (CASENT0790834; OKENT0019998; OKENT0019999; OKENT0020000, all in OIST).

##### Virtual dataset.

Volumetric raw data (in DICOM format), 3D rotation video (in .mp4 format, see Suppl. material 8: Video 6), still images of surface volume rendering, and 3D surface (in PLY format) of a non-type specimen (CASENT0790834) in addition to montage photos illustrating head in full-face view, profile and dorsal views of the body. The data is deposited at Dryad (Staab et al. 2018, http://dx.doi.org/10.5061/dryad.h6j0g4p) and can be freely accessed as virtual representation of the species. In addition to the data at Dryad, we also provide a freely accessible 3D surface model at Sketchfab (https://skfb.ly/6txNO).

##### Diagnosis.

*Proceratium japonicum* differs from the other east Asian members of the *P. silaceum* clade by the following character combination: medium-sized species (WL 0.72–1.00); sides of head convex, broadest above the level of eyes; anterior clypeal margin not protruding and slightly notched; frontal carinae well developed and widely separated, with large lamellae that extend laterally above the antennal insertions and reach posteriorly almost to the level of eyes; frontal furrow strongly developed; petiole squamiform, in profile not or only weakly narrowing dorsally, the base as or almost as broad as the apex, in dorsal view relatively narrow (DPeI <150); subpetiolar process developed, subtriangular, directing backwards; sculpture not deeply impressed, on abdominal segment III granulate and relatively regular; in addition to dense pubescence, some suberect to erect hairs present on scapes and dorsal surface of body.

##### Distribution and ecology.

This species is common from Japan (except Hokkaido) to Taiwan and usually collected in forests of relatively low elevation. It has also been reported from Yunnan Province in China. Thus, it is not unlikely that more records from the southern and eastern Chinese mainland will appear in the future if sampling effort is increased. No direct biological observations from China are available. In Japan, nests are typically found in deadwood in evergreen broadleaved forest (Onoyama and Yoshimura 2002). Colony size can reach over 150 workers and larval haemolymph feeding has been observed (Masuko 1986).

##### Taxonomic notes.

According to Baroni Urbani and de Andrade (2003) *P. japonicum* is most similar to *P. numidicum* Santschi, 1912, which is, however, a geographically widely separated species occurring in the eastern Mediterranean and northern Africa. We were not able to examine *P. japonicum* material from China. In Japan, specimens from the Ryukyu and Yaeyama islands are smaller than from the main islands (Onoyama and Yoshimura 2002, Baroni Urbani and de Andrade 2003), explaining the relatively large variation in body size.

From *P. longigaster*, the only other *P. silaceum* clade species in China and east Asia, *P. japonicum* can be separated by the shape of the petiole in profile that not or only weakly narrows dorsally (clearly narrowing dorsally, broader on the base than on the apex in *P. longigaster*). Also, the petiole in dorsal view is narrower in *P. japonicum* (DPeI <150) than in *P. longigaster* (DPeI ≥155). Furthermore, the frontal carinae in *P. japonicum* reach posteriorly almost to the level of eyes (shorter and ending well below the level of eyes in *P. longigaster*). *Proceratium japonicum* has only relatively few suberect to erect hairs that protrude from the dense pubescence on the dorsal body; those hairs are straight (never shaggy) and do not conspicuously project from LT3 over the constriction between LT3 and LT4 (many shaggy hairs projecting in *P. longigaster*); if single longer hairs are present, then they are not shaggy.

#### Proceratium longigaster

Taxon classificationAnimaliaORDOFAMILIA

Karavaiev, 1935

**Figure 20.**
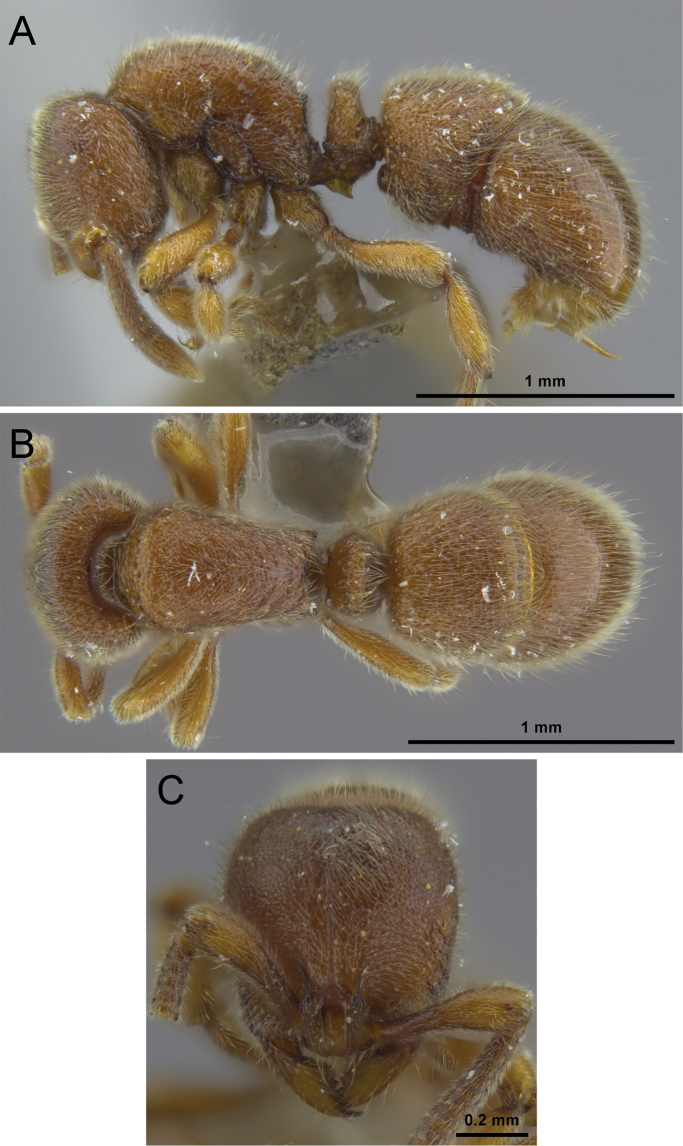
*Proceratium longigaster* non-type worker (CASENT0790673). **A** Body in profile **B** Body in dorsal view **C** Head in full-face view.

**Figure 21.**
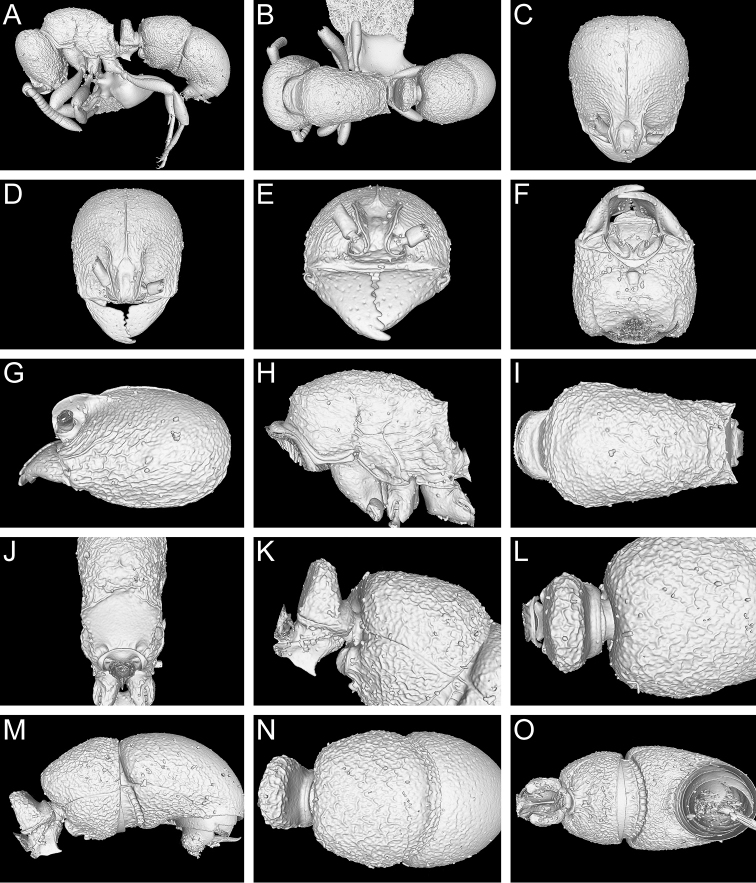
Still images from surface display volume renderings of 3D model of *Proceratium longigaster* non-type worker (CASENT0790673). **A** Body in profile **B** Body in dorsal view **C** Head in dorsal view **D** Head in anterodorsal view **E** Head in anterior view **F** Head in ventral view **G** Head in profile **H** Mesosoma in profile **I** Mesosoma in dorsal view **J** Propodeum in posterodorsal view **K** Abdominal segment II and parts of III in profile **L** Abdominal segment II and parts of III in dorsal view **M** Abdominal segments II–VII in profile **N** Abdominal segment III and parts of II and IV in dorsal view **O** Abdominal segments II–VII in ventral view.

Figs 2A , 20 , 21 , 25

Proceratium longigaster Karavaiev, 1935: 59 (w.), Vietnam (see also Xu 2000: 436, Baroni Urbani and de Andrade 2003: 438)

##### Type material.

**Holotype.** VIETNAM, Central Annam, close to Tourane, Bana, 1400 m asl, 30-IX.1931, leg. K. Davydov (CASENT0916806, in SIZK) [images examined].

##### Non-type material examined.

CHINA, Zhejiang Province, Gutianshan National Nature Reserve, ca. 30 km NW of Kaihua, 29°15'3"N, 118°8'34"E, 890 m asl, secondary subtropical mixed forest, Winkler extraction of a rotten log, 27-IV-2015, leg. Merle Noack, all with label ‘MS1857’ (CASENT0790844 in CASC; CASENT0790673 and CASENT0790843 in SWFU; CASENT0790845 in BMNH; CASENT0790846 in ZMBH).

##### Virtual dataset.

Volumetric raw data (in DICOM format), 3D rotation video (in .mp4 format, see Suppl. material 9: Video 7), still images of surface volume rendering, and 3D surface (in PLY format) of a non-type specimen (CASENT0790673) in addition to montage photos illustrating head in full-face view, profile and dorsal views of the body. The data is deposited at Dryad (Staab et al. 2018, http://dx.doi.org/10.5061/dryad.h6j0g4p) and can be freely accessed as virtual representation of the species. In addition to the data at Dryad, we also provide a freely accessible 3D surface model at Sketchfab (https://skfb.ly/6txOA).

##### Diagnosis.

*Proceratium longigaster* differs from the other east Asian members of the *P. silaceum* clade by the following character combination: medium-sized species (WL 0.75–0.89); sides of head slightly convex, broadest directly above the level of eyes; anterior clypeal margin not protruding and slightly notched; frontal carinae well developed and widely separated, with large lamellae that extend laterally above the antennal insertions and reach posteriorly about half the distance to the level of eyes; frontal furrow strongly developed; petiole squamiform; in profile, narrowing dorsally, the base clearly broader than the apex; in dorsal view, relatively wide (DPeI ≥155); subpetiolar process developed, subtriangular, directing backwards and relatively acute; sculpture deeply impressed, on abdominal segment III irregularly granular to reticulate (more so on dorsum); very hairy species; in addition to dense pubescence, many appressed to erect hairs present on entire body; abundant, long appressed, shaggy hairs project from LT3 distinctly over the constriction between LT3 and LT4.

##### Worker measurements.

**(n=5).** TL 2.66–3.10; EL 0.03–0.04; SL 0.42–0.46; HL 0.65–0.70; HLM 0.71–0.92; HW 0.60–0.66; WL 0.75–0.89; MFeL 0.43–0.54; MTiL 0.35–0.42; MBaL 0.26–0.29; PeL 0.20–0.22; PeW 0.31–0.34; LT3 0.43–0.49; LS4 0.28–0.30; LT4 0.56–0.63; OI 5; CI 92–98; SI 65–66; MFeI 71–83; MTiI 58–65; MBaI 42–45; DPeI 155–157; IGR 0.47–0.50; ASI 123–138.

##### Distribution and ecology.

The type locality is at ca. 1400 m asl in the Bà Nà hills close to Đà Nẵng city (referred to as Tourane in the original description), central Vietnam. The species is also known form Nangongshan Mountain, Mengla County, Yunnan Province (Xu 2000) (1525 m asl) and from Hunan Province (Guénard and Dunn 2012). In the places where it is known, specimens were collected from the ground in evergreen broadleaved forest. The new record from the Gutianshan National Nature Reserve, Zhejiang Province, is no exception in being from the same forest type albeit at lower elevation (890 m asl) and marks the easternmost distribution of the species. Thus, *P. longigaster* seems to be widespread in suitable forest habitats in south and east China and adjacent countries. No direct observations of biology and natural history are available.

##### Taxonomic notes.

This is a poorly known species. Since the single type specimen was not available for examination, Baroni Urbani and de Andrade (2003) were unable to formally treat it in their monograph. Karavaiev’s (1935) type specimen is lodged in the Schmalhausen Institute of Zoology (Kiev, Ukraine) and cannot be obtained as a loan. Fortunately, though, it has recently been imaged and the montage photos are available on AntWeb (CASENT0916806). Our new specimens agree with the type and the accounts of Xu (2000). Thus, with a note of caution, we feel confident enough to treat the specimens from Zhejiang Province as *P. longigaster*.

The only other *P. silaceum* clade species known from China and east Asia is *P. japonicum*, from which *P. longigaster* can be separated by the shape of the petiolar node, the frontal carinae, and the pilosity, among other characters (see the accounts for *P. japonicum* above).

#### Proceratium stictum

Taxon classificationAnimaliaORDOFAMILIA

clade

##### Definition.

Worker of this clade can be separated from all other *Proceratium* by the combination of calcar of strigil with a basal spine and clypeus distinctly and broadly notched (definition follows Baroni Urbani and de Andrade 2003).

##### Comments.

This is an exclusively tropical clade with species occurring in Africa, Australia, Madagascar, the Mascarene Islands, Mesoamerica, and tropical southeast Asia. Eleven extant species are known, of which *P. deelemani* Perrault, 1981, *P. foveolatum* Baroni Urbani & de Andrade, 2003, *P. stictum* Brown, 1958, and the newly described *P. shohei* are known from the oriental zoogeographic region. *Proceratium shohei* is the only species known from China.

#### Proceratium shohei

Taxon classificationAnimaliaORDOFAMILIA

Staab, Xu & Hita Garcia sp. n.

http://zoobank.org/09C9335F-C01F-4B6A-B289-1E6B9611E96F

**Figure 22.**
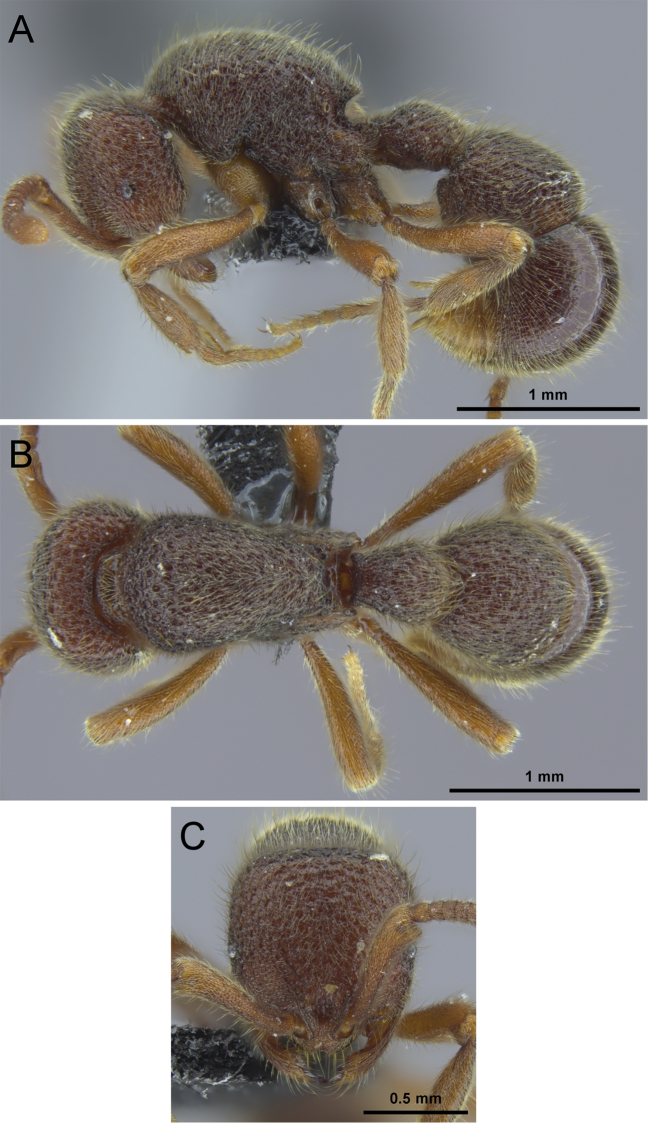
*Proceratium shohei* **sp. n.** holotype worker (CASENT0717686). **A** Body in profile **B** Body in dorsal view **C** Head in full-face view.

**Figure 23.**
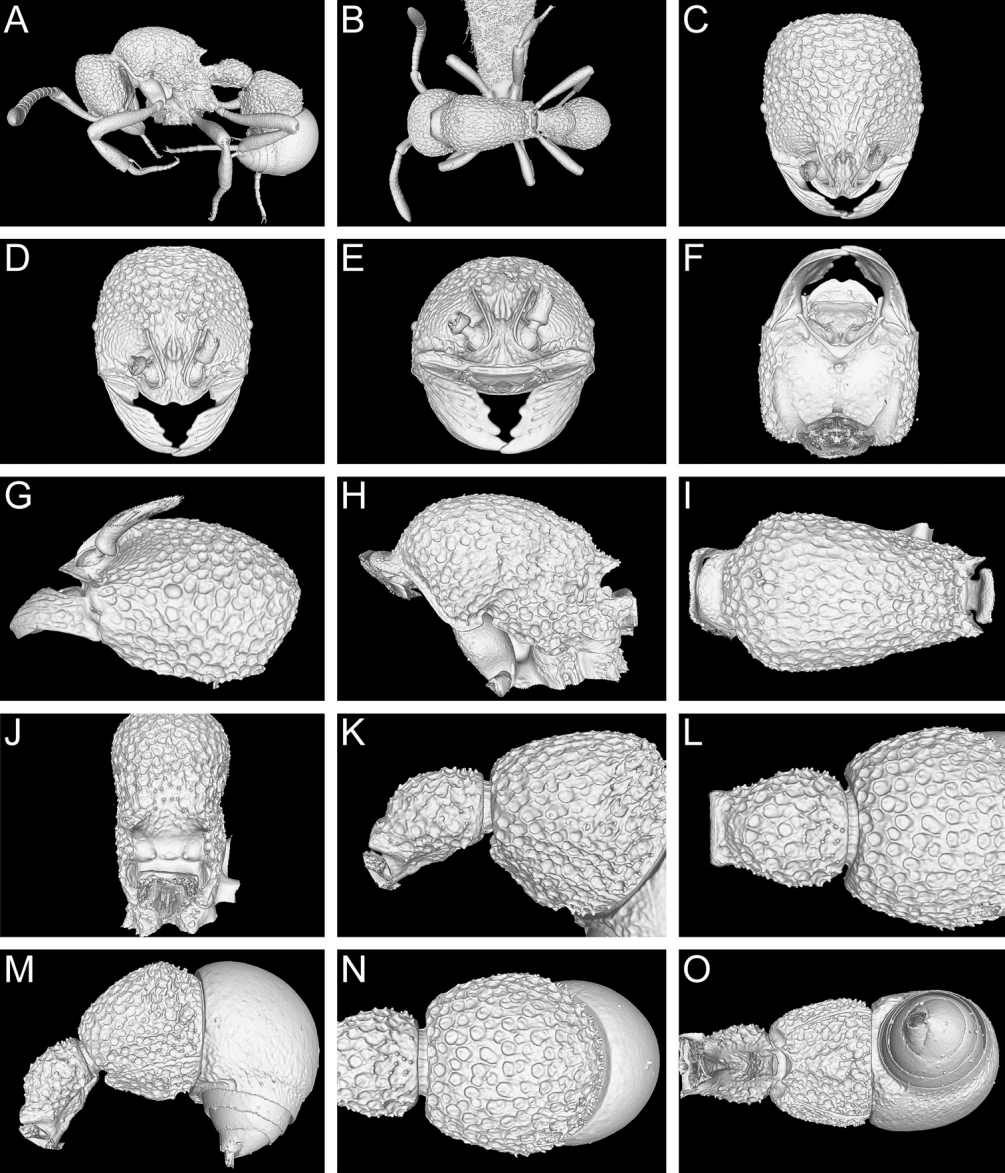
Still images from surface display volume renderings of 3D model of *Proceratium shohei* **sp. n.** holotype worker (CASENT0717686). **A** Body in profile **B** Body in dorsal view **C** Head in dorsal view **D** Head in anterodorsal view **E** Head in anterior view **F** Head in ventral view **G** Head in profile **H** Mesosoma in profile **I** Mesosoma in dorsal view **J** Propodeum in posterodorsal view **K** Abdominal segment II and parts of III in profile **L** Abdominal segment II and parts of III in dorsal view **M** Abdominal segments II–VII in profile **N** Abdominal segment III and parts of II and IV in dorsal view **O** Abdominal segments II–VII in ventral view.

Figs 3A , 3C , 22 , 23 , 25

##### Type material.

**Holotype.** Pinned worker from CHINA, Yunnan Province, Xishuangbanna, Kilometer 55 station, 21.964°N / 101.202°E, 820 m asl, rain forest, Winkler leaf litter extraction, 13-VI-2013, leg. Benoit Guénard, Benjamin Blanchard & Cong Liu, label ‘#05121’ (CASENT0717686), deposited in SWFU.

**Cybertype.** Volumetric raw data (in DICOM format), 3D rotation video (in mp4 format, see Suppl. material 10: Video 8), still images of surface volume rendering, and 3D surface (in PLY format) of the physical holotype (CASENT0717686) in addition to montage photos illustrating head in full-face view, profile and dorsal views of the body. The data is deposited at Dryad (Staab et al. 2018, http://dx.doi.org/10.5061/dryad.h6j0g4p) and can be freely accessed as virtual representation of the type. In addition to the cybertype data at Dryad, we also provide a freely accessible 3D surface model of the holotype at Sketchfab (https://sketchfab.com/models/0dd8217041274f268fae8897958d9b6a).

##### Diagnosis.

*Proceratium shohei* differs from the other oriental members of the *P. stictum* clade by the following character combination: head broadest at the level of eyes, sides and vertex of head weakly convex, almost straight; scapes relatively long (SI 72); frontal carinae relatively broad and slightly convex; posterodorsal corners of propodeum with broad teeth that project over less than half of the propodeal lobes in profile; petiole in dorsal view longer than broad; petiolar node relatively compressed dorsoventrally, subpetiolar process inconspicuous, a lamellae only, without a projection; LS3 with a straight ventral outline; abdominal segment IV strongly recurved and broadly rounded, LS4 reduced (IGR not measurable); head, mesosoma, petiole, and LT3 foveolate; LT4 smooth and shiny, dorsally without sculpture, laterally superficially punctured.

##### Worker measurements.

**Holotype.** TL 4.15; EL 0.09; SL 0.71; HL 0.99; HLM 1.13; HW 0.89; WL 1.25; MFeL 0.89; MTiL 0.71; MBaL 0.56; PeL 0.47; PeW 0.44; LT3 0.64; LS4 n.a.; LT4 0.66; OI 10; CI 90; SI 72; MFeI 100; MTiI 80; MBaI 63; DPeI 94; IGR n.a.; ASI 103.

##### Worker description.

In full-face view, head slightly longer than broad (CI 90), sides and vertex weakly convex, almost straight. Clypeus relatively broad, surrounding antennal insertions and protruding anteriorly, anterior clypeal margin with a distinct notch. Frontal carinae relatively short, broadly separated from each other, constantly diverging posteriorly and not covering antennal insertions, lateral expansions of frontal carinae slightly concave in full-face view; frontal area convex; frontal furrow absent. Genal carinae strongly developed; ventral face of head (gular area) concave. Eyes relatively large (OI 10), consisting of one convex ommatidium, located slightly anterior to the midline of head. Antennae 12-segmented, scapes comparatively long (SI 72), not reaching posterior head margin and thickening apically. Mandibles elongate and triangular, masticatory margin with three teeth in total, apical tooth large and acute, the other teeth smaller and decreasing in size from second to third tooth that is followed by a series of minute blunt denticles.

Mesosoma in profile convex and longer than maximum head length including mandibles. Lower mesopleurae (katepisterna) with demarcated sutures, upper mesopleurae (anepisterna), and promesonotum with inconspicuous and very shallow sutures; lower mesopleurae inflated posteriorly; posterodorsal corners of propodeum with broad teeth that project over less than half of the propodeal lobes in profile, propodeal lobes strongly developed as broadly triangular teeth protruding dorsolaterally; propodeal declivity almost vertical, slightly inclined anteriorly; in posterodorsal view, sides of propodeum separated from declivity by lamellate margins; propodeal spiracle relatively small, located above mid height; in profile, opening ellipsoid and facing posteriorly. Legs comparatively long; all tibiae with a pectinate spur; calcar of strigil with a basal spine; pretarsal claws simple; arolia present.

Petiole in dorsal view longer than broad, sides consistently diverging posteriorly, anterior border with a thick margin that is distinctly angulate on each side; in profile, petiolar node relatively compressed dorsoventrally, its anterior face slightly sloping; dorsum of node relatively flat, weakly convex; ventral face inconspicuous with a thin lamella and no projection.

In dorsal view, abdominal segment III anteriorly much broader than petiole, its sides weakly convex; abdominal sternite III extended ventrally, its outline straight, anteriomedially with a conspicuous depression marked by a broad rim. Constriction between abdominal segments III and IV deep. Abdominal segment IV very large, very strongly recurved (abdominal sternum IV reduced and IGR not measurable) and posteriorly rounded, with a thin lamella on its anterior border; abdominal tergum IV slightly longer than abdominal tergum III (ASI 103), remaining abdominal tergites and sternites inconspicuous and projecting anteriorly. Sting large and extended.

Whole body covered with dense relatively short decumbent to erect hairs; additionally significantly longer suberect to erect hairs abundant on the whole body, including legs and scapes; such hairs also present on funicular joints, but shorter and relatively thicker; dense appressed to decumbent pubescence on the funiculus only; mandibles striate; head, mesosoma, petiole, and abdominal segment III foveolate with superimposed punctures and granules, the foveae relatively deep, large, and irregular; abdominal segment IV smooth and shiny, dorsally without sculpture, laterally superficially punctured; scapes and legs densely punctured. Body color uniformly dark ferruginous-brown, antennae, legs, and abdominal segments V–VII orange brown.

##### Etymology.

This species is named in honor of Dr. Shohei Suzuki (1979–2016), a Japanese marine biologist whose life was tragically lost in a diving accident while conducting coral reef research in Okinawa.

##### Distribution and ecology.

No direct observations of biology and natural history are available. The type specimen was collected from rain forest leaf litter. Like many other ant species occurring in the tropical rain forest of Xishuangbanna, the species probably also occurs in adjacent countries such as Laos or Thailand.

**Figure 24.**
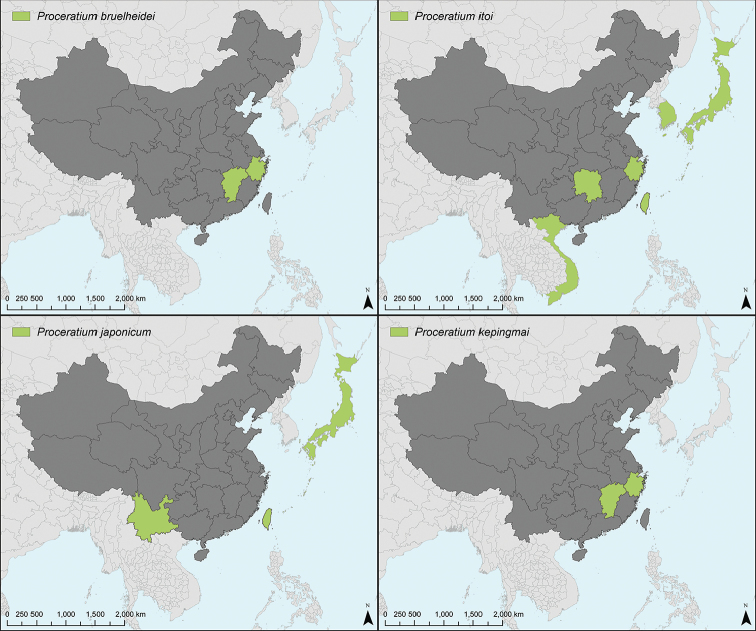
Maps of China (country is shown in dark grey with highlighted country and province borders) and South East Asia displaying known species distribution ranges (in green) of *P. bruelheidei*, *P. itoi*, *P. japonicum*, and *P. kepingmai*.

**Figure 25.**
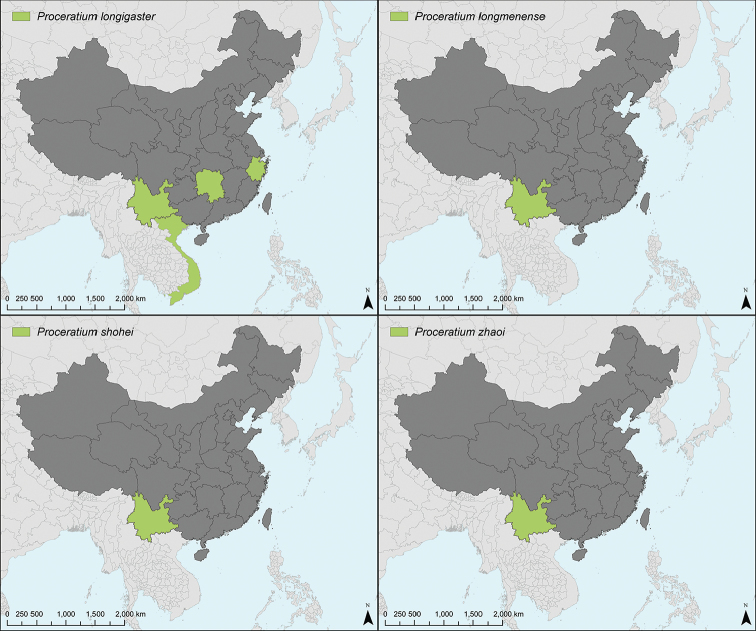
Maps of China (country is shown in dark grey with highlighted country and province borders) and South East Asia displaying known species distribution ranges (in green) of *P. longigaster*, *P. longmenense*, *P. shohei*, and *P. zhaoi*.

##### Taxonomic notes.

In Liu et al. (2015b) this species was erroneously listed as *P. deelemani*, a species known from Borneo, peninsular Malaysia, and Thailand (see Baroni Urbani and de Andrade 2003). However, a careful reexamination of the specimen from Yunnan and comparisons with images of the holotype of *P. deelemani* (CASENT0915370) and further *P. deelemani* specimens from Borneo (CASENT0790842, CASENT0790847, CASENT0790848; see Suppl. material 2: Figure S1 for micro CT images of CASENT0790842 and see Suppl. material 11: Video 9 for a 3D rotation video of the same specimen) revealed considerable morphological differences that convinced us to separate both species and to describe *P. shohei* as new. Among the other species of the *P. stictum* clade occurring in the oriental zoogeographic region (*P. deelemani*, *P. foveolatum*, *P. stictum*), *P. shohei* is unsurprisingly most similar to *P. deelemani*, but both species can be safely and easily separated. *Proceratium shohei* has an indistinct subpetiolar process without a median anterior projection (subpetiolar process with a distinct tooth in *P. deelemani*; opposed to the *P. itoi* clade, the subpetiolar process is an informative character in the *P. stictum* clade). Also, *P. shohei* has relatively longer scapes (SI 72) (SI 58–68 in *P. deelemani*), the posterodorsal corner of the propodeum with relatively shorter teeth that project over less than half of the length of the propodeal lobes in profile (at least projecting over half of propodeal lobes in *P. deelemani*), a very reduced LS4 so that IGR cannot be measured (LS4 also reduced but IGR 0.23–0.29 in *P. deelemani*), a straight ventral outline of LS3 (with a depression in *P. deelemani*), and slightly convex frontal carinae (slightly concave in *P. deelemani*). Superficially, *P. shohei* also resembles *P. stictum* and *P. foveolatum*. From *P. stictum* it can be distinguished by the subpetiolar process without a median anterior projection (subpetiolar process with a distinct tooth in *P. stictum*), the longer teeth on the posterodorsal corners of the propodeum that project straightly backwards (short and blunt, projecting slightly dorsally in *P. stictum*), and the foveolate sculpture of the head, mesosoma, petiole, and LT3 (coarsely granulate with superimposed fovea in *P. stictum*). The sculpture of the integument likewise easily distinguishes *P. shohei* from *P. foveolatum*, which has the entire integument including LT4 covered with large, deep, regular, and clearly demarcated fovea (fovea smaller and shallower, at most superficial punctures but no fovea on LT4 in *P. shohei*). Also, in *P. foveolatum* LT4 is extended posteriorly and forms a broad, strong angle while LT4 is not as extended and broadly rounded in *P. shohei*.

##### Variation.

Since this species is known only from the holotype there is no available information about intraspecific variation.

## Discussion

### The genus *Proceratium* in China

As for most other regions in which *Proceratium* occur, collection records and distributional information for the Chinese fauna is very limited, which is likely a consequence of the species’ cryptobiotic and partly subterranean lifestyle. This is especially true for the *P. itoi* clade that based on currently available information seems to be restricted to east and southeast Asia (Baroni Urbani and de Andrade 2003, Guénard et al. 2017). All species of this clade except *P. malesianum* (Peninsular Malaysia) and *P. williamsi* (Buthan; India) have been recorded from China but are generally only known from few locations. Further collections targeting leaf litter and soil (Wong and Guénard 2017) will be necessary to clarify species-specific distribution ranges. It is expected that several species of the genus, of which some might also be new to science, occur in the large areas in south and southeast China that lack records so far (Guénard et al. 2017). Increased specimen availability will also allow associating queens and males to workers, as both reproductive castes are only known for *P. itoi* and *P. japonicum* (Onoyama and Yoshimura 2002, Baroni Urbani and de Andrade 2003), while for *P. zhaoi* queens have been described (Xu 2000).

Recently, Liu et al. (2015b) recorded *P. deelemani* Perrault, 1981, a conspicuous large-bodied species originally described from Borneo, from the tropical rain forests of Xishuangbanna, Yunnan Province. After careful reexamination of the single available specimen, we find that this species differs in several important characters from *P. deelemani* and describe it as *P. shohei*. The species belongs to the *P. stictum* clade and represents the northernmost record of this tropical clade in Asia.

With the exception of *P. bruelheidei*, which type habitat is an early successional tree plantation with relatively open soil and comparatively little litter cover, all other Chinese species have only been collected from old-growth forests. Unfortunately, forests in tropical and subtropical China have been heavily transformed and fragmented (e.g. Zhang and Song 2006, Li et al. 2009), which has largely unknown but likely negative consequences for native ant assemblages (e.g. Liu et al. 2016).

Direct observations of ecology and natural history are very rare for Chinese *Proceratium*. To the best of our knowledge, the nest size of 45 individuals for the type colony of *P. zhaoi* given by Xu (2000) is the only published information on that matter. We assume that the general natural history of the Chinese species conforms to the observations from other parts of the world outlined above (Baroni Urbani and de Andrade 2003). This life history is also documented for the Japanese populations of *P. japonicum* and *P. itoi* (Masuko 1986, Onoyama and Yoshimura 2002), two species that occur in China. As for distribution ranges and habitat preferences, further observations and collections will be necessary to extend our knowledge on natural history.

### Microtomography

One problem encountered by Hita Garcia et al. (2017b) was the poor recovery of pilosity in the 3D reconstructions due to insufficient voxel resolution, which was resolved in Hita Garcia et al. (2017a) by scanning single body parts at higher resolutions. Nevertheless, in this study, we aimed to turn this handicap into an advantage. Like most proceratiines, the Chinese species of *Proceratium* are very hairy and covered in a thick pelt, which makes morphological examinations challenging. The furry coats cover and hide important character states, such as surface sculpture, and, to make things worse, many specimens are extremely dirty due to numerous soil particles caught within the hairs. Furthermore, potentially harmful cleaning or dissections of specimens are out of the question since, as is typical for the genus in general, the available Chinese material is way too scarce and valuable.

By applying micro-CT scanning and virtually “shaving” the specimens, we were able to examine proceratiine morphology in more detail resulting in clearer diagnostic character definitions and species delimitations without causing any physical harm to the few available specimens. This approach might also be useful for morphological examinations of other very hairy groups of ants, such as *Discothyrea* Roger or many species of *Tetramorium* Mayr (previously grouped in the genus *Triglyphothrix* Forel). For those ants it could complement high-resolution montage images illustrating specimens including pilosity and hairs, which can be diagnostic characters and useful for species identifications, as illustrated with the present study.

## Supplementary Material

XML Treatment for Proceratium itoi

XML Treatment for Proceratium bruelheidei

XML Treatment for Proceratium itoi

XML Treatment for Proceratium kepingmai

XML Treatment for Proceratium longmenense

XML Treatment for Proceratium zhaoi

XML Treatment for Proceratium silaceum

XML Treatment for Proceratium japonicum

XML Treatment for Proceratium longigaster

XML Treatment for Proceratium stictum

XML Treatment for Proceratium shohei

## Supplementary materials

Supplementary material 1 Table S1 Data type: species data Explanation note: Overview of specimens of non-Chinese Proceratium species examined in this study. This is an open access article distributed under the terms of the Creative Commons Attribution License (CC BY 4.0), which permits unrestricted use, distribution, and reproduction in any medium, provided the original author and source are credited.Michael Staab, Francisco Hita Garcia, Cong Liu, Zheng-Hui Xu, Evan P. Economo

Supplementary material 2 Figure S1. Data type: multimedia Explanation note: Still images from surface display volume renderings of 3D model of *Proceratium deelemani* non-type worker (CASENT0790842). **A** Body in profile **B** Body in dorsal view **C** Head in dorsal view **D** Head in anterodorsal view **E** Head in anterior view **F** Head in ventral view **G** Head in profile **H** Mesosoma in profile **I** Mesosoma in dorsal view **J** Propodeum in posterodorsal view **K** Abdominal segment II and parts of III in profile **L** Abdominal segment II and parts of III in dorsal view **M** Abdominal segments II–VII in profile **N** Abdominal segment III and parts of II and IV in dorsal view **O** Abdominal segments II–VII in ventral view. This is an open access article distributed under the terms of the Creative Commons Attribution License (CC BY 4.0), which permits unrestricted use, distribution, and reproduction in any medium, provided the original author and source are credited.Michael Staab, Francisco Hita Garcia, Cong Liu, Zheng-Hui Xu, Evan P. Economo

Supplementary material 3 Video 1 Data type: (Video file (.mp4)) Explanation note: *Proceratium bruelheidei* sp. n. holotype worker (CASENT0790023). 3D rotation video based on volumetric surface rendering of full body.. This is an open access article distributed under the terms of the Creative Commons Attribution License (CC BY 4.0), which permits unrestricted use, distribution, and reproduction in any medium, provided the original author and source are credited.Author: Francisco Hita Garcia

Supplementary material 4 Video 2 Data type: (Video file (.mp4)) Explanation note: *Proceratium itoi* non-type worker (OKENT0016142). 3D rotation video based on volumetric surface rendering of full body. This is an open access article distributed under the terms of the Creative Commons Attribution License (CC BY 4.0), which permits unrestricted use, distribution, and reproduction in any medium, provided the original author and source are credited.Author: Francisco Hita Garcia

Supplementary material 5 Video 3 Data type: (Video file (.mp4)) Explanation note: *Proceratium kepingmai* sp. n. holotype worker (CASENT0790031). 3D rotation video based on volumetric surface rendering of full body. This is an open access article distributed under the terms of the Creative Commons Attribution License (CC BY 4.0), which permits unrestricted use, distribution, and reproduction in any medium, provided the original author and source are credited.Author: Francisco Hita Garcia

Supplementary material 6 Video 4 Data type: (Video file (.mp4)) Explanation note: *Proceratium zhaoi* paratype worker (CASENT0790671). 3D rotation video based on volumetric surface rendering of full body. This is an open access article distributed under the terms of the Creative Commons Attribution License (CC BY 4.0), which permits unrestricted use, distribution, and reproduction in any medium, provided the original author and source are credited.Author: Francisco Hita Garcia

Supplementary material 7 Video 5 Data type: (Video file (.mp4)) Explanation note: *Proceratium nujiangense* syn. n. paratype worker (CASENT0790672). 3D rotation video based on volumetric surface rendering of full body. This is an open access article distributed under the terms of the Creative Commons Attribution License (CC BY 4.0), which permits unrestricted use, distribution, and reproduction in any medium, provided the original author and source are credited.Author: Francisco Hita Garcia

Supplementary material 8 Video 6 Data type: (Video file (.mp4)) Explanation note: *Proceratium japonicum* non-type worker (CASENT0790834). 3D rotation video based on volumetric surface rendering of full body. This is an open access article distributed under the terms of the Creative Commons Attribution License (CC BY 4.0), which permits unrestricted use, distribution, and reproduction in any medium, provided the original author and source are credited.Author: Francisco Hita Garcia

Supplementary material 9 Video 7 Data type: (Video file (.mp4)) Explanation note: *Proceratium longigaster* non-type worker (CASENT0790673). 3D rotation video based on volumetric surface rendering of full body. This is an open access article distributed under the terms of the Creative Commons Attribution License (CC BY 4.0), which permits unrestricted use, distribution, and reproduction in any medium, provided the original author and source are credited.Author: Francisco Hita Garcia

Supplementary material 10 Video 8 Data type: (Video file (.mp4)) Explanation note: Proceratium shohei sp. n. holotype worker (CASENT0717686). 3D rotation video based on volumetric surface rendering of full body. This is an open access article distributed under the terms of the Creative Commons Attribution License (CC BY 4.0), which permits unrestricted use, distribution, and reproduction in any medium, provided the original author and source are credited.Author: Francisco Hita Garcia

Supplementary material 11 Video 9 Data type: (Video file (.mp4)) Explanation note: *Proceratium deelemani* non-type worker (CASENT0790842). 3D rotation video based on volumetric surface rendering of full body. This is an open access article distributed under the terms of the Creative Commons Attribution License (CC BY 4.0), which permits unrestricted use, distribution, and reproduction in any medium, provided the original author and source are credited.Author: Francisco Hita Garcia
