# Supplementary figures and images for: Systematics of the ant genus Proceratium Roger (Hymenoptera, Formicidae, Proceratiinae) in China – with descriptions of three new species based on micro-CT enhanced next-generation-morphology
Source: Zookeys. 2018 Jun 4;(770):137–92. doi: 10.3897/zookeys.770.24908 (PMC6041363; doi:10.3897/zookeys.770.24908)

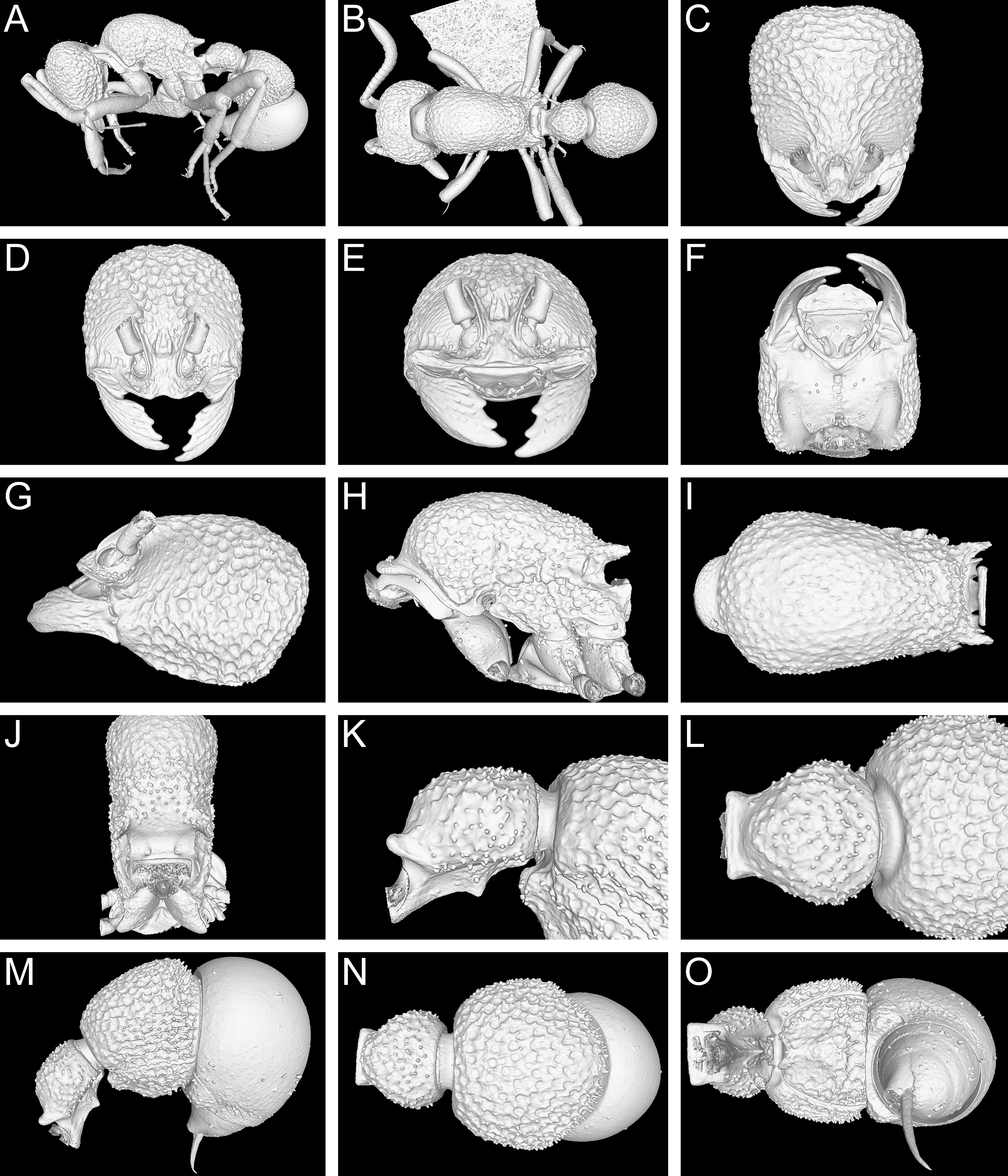

Supplement: Supplementary material 2 — Figure S1. [file zookeys-770-137-s002.tif]
